# Clinical, radiological, and molecular insights into extracranial metastases from adult gliomas

**DOI:** 10.1093/neuonc/noaf178

**Published:** 2025-08-16

**Authors:** Julie Jacobsen, Alessio Locallo, Colm J O’Rourke, Jonathan F Carlsen, Jonathan Cohen, Jesper D Ewald, David Scheie, Kirsten Grunnet, Ane Y Schmidt, Linea C Melchior, Vibeke A Larsen, Jesper B Andersen, Hans S Poulsen, Joachim Weischenfeldt, Helle Broholm, Signe R Michaelsen, Bjarne W Kristensen

**Affiliations:** Biotech Research and Innovation Centre (BRIC), Department of Health and Medical Sciences, University of Copenhagen, Copenhagen, Denmark; Department of Pathology, The Bartholin Institute, Rigshospitalet, Copenhagen University Hospital, Copenhagen, Denmark; Finsen Laboratory, Rigshospitalet, Copenhagen University Hospital, Copenhagen, Denmark; Biotech Research and Innovation Centre (BRIC), Department of Health and Medical Sciences, University of Copenhagen, Copenhagen, Denmark; Biotech Research and Innovation Centre (BRIC), Department of Health and Medical Sciences, University of Copenhagen, Copenhagen, Denmark; Department of Clinical Medicine, University of Copenhagen, Copenhagen, Denmark; Department of Radiology, Rigshospitalet, Copenhagen University Hospital, Copenhagen, Denmark; Department of Radiology, Rigshospitalet, Copenhagen University Hospital, Copenhagen, Denmark; Department of Clinical Research, University of Southern Denmark, Odense, Denmark; Department of Pathology, Odense University Hospital, Odense, Denmark; Department of Pathology, Rigshospitalet, Copenhagen University Hospital, Copenhagen, Denmark; Department of Clinical Medicine, University of Copenhagen, Copenhagen, Denmark; The DCCC Brain Tumor Center, Department of Oncology, Rigshospitalet, Copenhagen University Hospital, Copenhagen, Denmark; Center for Genomic Medicine, Rigshospitalet, Copenhagen University Hospital, Copenhagen, Denmark; Department of Pathology, Rigshospitalet, Copenhagen University Hospital, Copenhagen, Denmark; Department of Radiology, Rigshospitalet, Copenhagen University Hospital, Copenhagen, Denmark; Biotech Research and Innovation Centre (BRIC), Department of Health and Medical Sciences, University of Copenhagen, Copenhagen, Denmark; The DCCC Brain Tumor Center, Department of Oncology, Rigshospitalet, Copenhagen University Hospital, Copenhagen, Denmark; Finsen Laboratory, Rigshospitalet, Copenhagen University Hospital, Copenhagen, Denmark; Biotech Research and Innovation Centre (BRIC), Department of Health and Medical Sciences, University of Copenhagen, Copenhagen, Denmark; Department of Pathology, Rigshospitalet, Copenhagen University Hospital, Copenhagen, Denmark; The DCCC Brain Tumor Center, Department of Oncology, Rigshospitalet, Copenhagen University Hospital, Copenhagen, Denmark; Biotech Research and Innovation Centre (BRIC), Department of Health and Medical Sciences, University of Copenhagen, Copenhagen, Denmark; Department of Pathology, The Bartholin Institute, Rigshospitalet, Copenhagen University Hospital, Copenhagen, Denmark; The DCCC Brain Tumor Center, Department of Oncology, Rigshospitalet, Copenhagen University Hospital, Copenhagen, Denmark; Department of Clinical Medicine, University of Copenhagen, Copenhagen, Denmark; Biotech Research and Innovation Centre (BRIC), Department of Health and Medical Sciences, University of Copenhagen, Copenhagen, Denmark; Department of Pathology, The Bartholin Institute, Rigshospitalet, Copenhagen University Hospital, Copenhagen, Denmark

**Keywords:** Glioblastoma, glioma, gliosarcoma, metastasis, TME

## Abstract

**Background:**

Extracranial metastases from adult gliomas cause diagnostic and therapeutic challenges and are generally poorly investigated. The aim of this study was to provide clinical and molecular insights into glioma metastasis.

**Methods:**

Our cohort comprised tumor tissue from 16 glioma patients with metastasis (14 glioblastomas and 2 lower-grade gliomas). Paired primary tumors, recurrences, and metastases were investigated by DNA sequencing, genome-wide DNA methylation profiling, RNA sequencing, immunohistochemistry, and MRI examinations.

**Results:**

The metastases were distributed across the scalp/upper neck (8), lymph nodes (5), bone (2), and liver (1). Six out of 14 glioblastomas displayed significant sarcomatous differentiation, consistent with the otherwise rare histological subtype gliosarcoma. A majority of the scalp lesions were connected to an intracranial brain tumor via tumor extension through craniotomy burr holes, proposing that surgery is a contributing factor to tumor spread. Next-generation sequencing-based mutational analysis revealed that the true metastases originated from the primary tumors and not later recurrences. We observed tumor plasticity as the tumors progressed to metastasis, demonstrated by changes in epigenetic methylation classes and transcriptional subtypes. Despite different locations of metastases in the cohort, the immune cell composition in the tumor microenvironment remained overall stable during tumor progression.

**Conclusion:**

Metastases from adult gliomas originate from the primary brain tumors and not later recurrences. While RNA sequencing and methylation profiling revealed tumor plasticity during progression to metastasis, the immune cell composition remained overall stable.

**Key points**

- Primary brain tumors are the clonal origin of glioma metastases.
- Switch in RNA subtypes and methylation classes occurs during tumor progression.
- The immune cell composition in the TME remains stable.

**Importance of the Study**

There is no efficient treatment approach for glioma patients developing extracranial metastases, and the rarity of this disease limits research in the area. Having established the largest cohort to date of metastasizing adult gliomas, we show that glioblastomas and lower-grade gliomas may metastasize to the scalp/neck, liver, lymph nodes, and bones. Our findings demonstrate that scalp lesions may result from tumor extension through craniotomy burr holes and that sarcomatous differentiation is common. Mutational analysis revealed that the true metastases originated from the primary tumors, and we observed a switch in transcriptional subtypes and methylation classes demonstrating tumor plasticity upon disease progression. Detection of similar immune cell compositions in paired metastatic lesions and primary brain tumors suggests that the immune privileged milieu in the brain is of less importance for shaping this microenvironment than previously thought. These finding may have future therapeutic implications for patients with metastatic glioblastomas.

Glioblastoma, IDH-wildtype, WHO grade 4 is the most common and malignant primary brain tumor in adults^1^ with a median overall survival of 14.6 months.^2^ Complete tumor resection and successful treatment is challenged by chemoresistance,^3^ infiltrative growth,^4^ and, in 0.4%-0.5% of the cases, development of extracranial metastasis.^5,6^ Although some studies have reported similar to decreased survival in these patients compared to patients without extracranial metastasis,^7,8^ knowledge about the clinical characteristics is lacking and the underlying biological mechanisms remain unsolved.

Physical barriers such as the blood-brain barrier and dura mater are believed to prevent tumor cell dissemination from brain tumors.^9^ However, previous studies have shown that 20%-39% of glioblastoma patients have circulating tumor cells,^10,11^ and there are reports of organ recipients developing metastasis following transplantation from donors with glioblastoma.^12^ It has been proposed that surgical interventions including neurosurgery and placement of ventricular peritoneal shunts may induce glioblastoma metastasis.^13^ Nonetheless, reports of metastasis with no prior surgical intervention^14,15^ demonstrate the metastatic capacity of brain tumors in this subgroup of poorly characterized patients.

In the existing literature, only a limited number of studies aiming to shed light on mechanisms involved in glioma metastasis use methods other than conventional immunohistochemistry (IHC). Typically, these studies are confined to tumor tissue from a single patient.^5,16–20^ While the use of genome-wide DNA methylation profiling enables the detection and characterization of rare and diagnostically challenging brain tumor entities,^21,22^ merely a few reports describe the implementation of this tool in the context of a potential glioma metastasis.^5,23^

The development of metastasis generally involves a complex interplay between different cell types in the tumor microenvironment (TME).^24^ As increased knowledge hereof may lead to the development of efficient therapies, developing a deep understanding of the immune microenvironment in glioma metastases is of critical importance. A pivotal example is the use of checkpoint inhibitors in melanoma patients, which has led to a significant increase in survival.^25^ Another aspect with potential clinical relevance in the TME is the presence of tumor stem-like cells which can generate new cell populations with metastatic potential,^26^ and promote both chemoresistance and recurrence in gliomas.^27^ Nevertheless, no effort has been made to characterize these aspects of the TME and their role in glioma metastasis.

Addressing the clear knowledge gaps in the biological mechanisms behind glioma metastasis, we present findings from the largest reported cohort of its kind, using cutting-edge methods rarely used in this context before. We utilized paired tumor tissue from a total of 16 patients with adult glioma metastasis, including 14 patients with glioblastoma and 2 patients with lower-grade glioma. Applying sequencing-based mutational analysis, genome-wide DNA methylation profiling, RNA sequencing, and artificial intelligence-based quantitation of TME IHC markers, we provide novel insights into the clonal evolution, and epigenetic, transcriptional, and TME dynamics of this rare disease trajectory.

## Materials and Methods

### Patient Cohort

The cohort initially comprised 20 patients, however, 2 patients were excluded due to a lack of tissue availability, and 2 patients were excluded due to clinical findings not supporting the development of true metastasis: seeding to the spinal fluid and spread along a cystoperitoneal shunt. The remaining 16 patients underwent surgical resection of a primary adult glioma at the Copenhagen University Hospital, Rigshospitalet, Copenhagen, Denmark between 1999 and 2019. The metastatic lesions were resected at various locations in the Zealand area. For patients 6 and 9, the metastatic lesions were detected and collected during autopsy. Sequential formalin-fixed paraffin-embedded (FFPE) samples were obtained from the initial surgery, tumor recurrence (if any), and from the metastatic lesion. If the patient experienced multiple intracranial recurrences, the latest recurrence was used. For patients 3 and 13, the first intracranial recurrence was used instead of the primary brain tumor, due to a lack of tissue availability. At the initiation of this study, the tumor specimens were re-evaluated by 2 neuropathologists (B.W.K. and D.S.) according to the WHO 2021 classification of brain tumors. If necessary, additional staining for glial fibrillary acidic protein (GFAP) and isocitrate dehydrogenase 1 (IDH1) R132H was performed. Based on magnetic resonance-imaging (MRI) scans, patients were annotated as having either a true, distant metastasis or an extracranial extension of the brain tumor into the scalp. Some patients had other suspected metastases detected on scans which were not included in this study as the tissue was not resected (Supplementary Table 1).

### Radiological Tumor Characterization

Brain MRIs were reviewed by a radiology resident (J.C.) and a neuroradiologist (J.F.C.). Primary brain scans (MRI of the primary brain tumor) and secondary brain scans (MRI at the time of metastasis or extracranial extension) were evaluated using contrast-enhanced *T*_1_, *T*_2_, and Fluid-Attenuated Inversion Recovery (FLAIR) sequences. For primary brain scans, contrast-enhancement pattern, presence of central necrosis, FLAIR/T2 mismatch, tumor mass effect, and tumor volume were registered. To identify potential routes of tumor dissemination, brain tumor distance to the dura mater, large vessels, and ventricles were evaluated. Examples of the different parameters are shown in Supplementary Figure 1. See Supplementary Methods for details.

### DNA Isolation for Sequencing and Genome-Wide DNA Methylation Profiling

DNA was extracted from 10 µm FFPE tumor sections. To increase tumor purity, larger areas of non-neoplastic tissue were subject to macrodissection supervised by a neuropathologist (B.W.K.). DNA was extracted using Proteinase K (cat.no: 19133, Qiagen) followed by a column-based polymerase chain reaction inhibitor removal protocol according to the manufacturer’s instructions (cat.no: D6030, Zymo Research). DNA concentration was measured using a Qubit 4 Fluorometer (Thermo Fisher, firmware version 2.10).

### Targeted DNA Sequencing

Targeted next-generation sequencing (NGS) was performed at the Department of Genomic Medicine, Rigshospitalet, Copenhagen University Hospital, Denmark using the Illumina TruSight Oncology 500 (TSO500) panel (information about which tumors were sequenced are given in Table 1). The protocol was performed according to the manufacturer’s instructions and paired-end sequencing was carried out on NovaSeq 6000 (Illumina). See Supplementary Methods for details.

**Table 1.** Cohort characteristics

ID	Sex	Age, debut	Diagnosis, primary tumor	*IDH1* status	MGMT promoter methylation status	Number of craniotomies	True metastasis	Extracranial extension	Treatment	OS, months	Survival from true metastasis/extracranial extension, days	Subject to NGS	Subject to methylation profiling	Subject to RNA sequencing
**1**	M	55	GBM	WT	Meth	1 (2 including autopsy)	+ (lymph node, colli sin.)	-	N/A	8	92	Primary tumor, lymph node	Primary tumor, lymph node	-
**3**	M	27	O2	Mut	Meth	3	+ (lymph node, colli dxt.)	-	P: None R1: None R2: RT M: TMZ, RT	147	211	First recurrence, second recurrence, lymph node	First recurrence, second recurrence, lymph node	-
**6**	M	29	A2	Mut	Meth	2 (3 including autopsy)	+ (columna)	-	P: None R1: RT	35	0 (Metastasis discovered postmortem)	Primary tumor, recurrent tumor, columna	Primary tumor, recurrent tumor, columna	-
**7**	M	46	GBM	WT	Unmeth	1	+ (lymph node, regio nuchae dxt.)	-	N/A	7	25	Primary tumor, lymph node	Primary tumor, lymph node	-
**8**	F	59	GBM	WT	Unmeth	1	+ (lymph nodes, mediastinum)	-	P: TMZ, RT M: Iri, Bev	7	28	Primary tumor, lymph node	Primary tumor, lymph node	Primary tumor, lymph node
**9**	M	61	GBM	WT	Meth	1	+ (liver)	-	None	5	0 (Metastasis discovered postmortem)	Primary tumor, liver	Primary tumor, liver	Primary tumor, liver
**12**	M	62	GBM (GS)	WT	Unmeth	2	+ (upper neck)	-	P: TMZ, RT R1: Bev, Iri EE: None	15	184	Primary tumor	Primary tumor, upper neck	Primary tumor, recurrent tumor, upper neck
**15**	M	54	GBM	WT	Unmeth	2	+ (columna)	-	P: TMZ, RT R1: None M: RT	7	31	Primary tumor, recurrent tumor, columna	Primary tumor, recurrent tumor, columna	-
**16**	M	54	GBM	WT	Unmeth	3	+ (scalp)	-	P: TMZ, RT R1: Iri, Bev M: None	12	32	Primary tumor	Primary tumor	-
**20**	F	58	GBM	WT	Unmeth	2	+ (lymph nodes, mediastinum)	-	P: TMZ, RT R1: TMZ M: None	18	15	Primary tumor, recurrent tumor, lymph nodes	Primary tumor, recurrent tumor, lymph nodes	-
**10**	F	62	GBM	WT	Unmeth	3	-	+ (scalp)	P: TMZ, RT R1: Bev, Lom R2: None EE: None	18	115	Primary tumor	Primary tumor, scalp	-
**11**	M	59	GBM (GS)	WT	Unmeth	1	-	+ (scalp)	P: TMZ, RT EE: None	20	177	Primary tumor	Primary tumor, scalp	-
**13**	M	40	GBM	WT	Unmeth	5	-	+ (scalp)	P: TMZ, RT R1: None R2: None R3: Sel, Bev, Lom R4: None EE: None	44	159	First recurrence	First recurrence, scalp	First recurrence, last recurrence, scalp
**17**	M	59	GBM	WT	Unmeth	3	-	+ (scalp)	P: TMZ, RT R1: None R2: None EE: None	13	54	Primary tumor	Primary tumor, scalp	Primary tumor, recurrent tumor, scalp
**18**	M	65	GBM	WT	Unmeth	2	-	+ (scalp)	P: TMZ, RT R1: Bev, Iri EE: RT	11	200	Primary tumor	Primary tumor, scalp	-
**19**	F	47	GBM (GS)	WT	Unmeth	4	-	+ (scalp)	P: TMZ, RT R1: Bev, Iri R2: None EE: None	17	86	Primary tumor	Primary tumor, scalp	Primary tumor, recurrent tumor, scalp

*Abbreviations:* M, male; F, female; GBM, glioblastoma; O2, oligodendroglioma grade 2; A2, astrocytoma grade 2; GS, gliosarcoma; *IDH1*, isocitrate dehydrogenase 1; Mut, mutated; WT, wildtype; *MGMT*, O6-methylguanine-DNA-methyltransferase; Meth, methylated; Unmeth, unmethylated; Sin, sinister; *Dxt*, dexter; N/A, not available; P, primary tumor; R, recurrent tumor; M, metastasis; EE, extracranial extension; TMZ, temozolomide; Iri, irinotecan; Bev, bevacizumab; Lom, lomustine; Sel, selinexor; OS, overall survival; NGS, next-generation sequencing.

After BWA-MEM alignment of raw FASTQ files and GATK preprocessing, somatic single nucleotide variants (SNVs) were called with MuTect2,^28,29^ stringently filtered (≥10× depth, ≥5 alt reads, variant allele frequency (VAF) > 5%; purity > 10% for TERT promoter) and functionally annotated. Tumor mutational burden (TMB) was computed as the ratio between nonsynonymous mutations and the nucleotide size of the capture kit (TSO500). Microsatellite instability was assessed using MSIsensor2 (https://github.com/niu-lab/msisensor2). Patients 1 and 6 were excluded during quality control (QC) (TMB and QC metrics are summarized in Supplementary Table 2). See Supplementary Methods for details.

Clonal phylogenies were derived by clustering driver SNVs that passed QC criteria (Supplementary Table 3) according to their cellular prevalence (Supplementary Figure 2), integrating large-scale copy number alterations (CNAs) from methylation profiles and surgical timing, with trunk/branch/leaf assignments. For patient 3, the first of 2 intracranial recurrences were used instead of the primary tumor due to lack of tissue availability. See Supplementary Methods for details.

### Genome-Wide DNA Methylation Profiling

Genome-wide DNA methylation profiling was carried out at Life & Brain GmbH, Platform Genomics in Bonn, Germany using the Illumina Infinium MethylationEPIC (850K) BeadChip (information about which tumors were profiled is given in Table 1). Output data were uploaded to the online DNA methylation-based CNS tumor classifier created by the German Cancer Research Center (DKFZ) and Heidelberg University.^30^ Using classifier version 12.5, we included all cases with a family- and class prediction score ≥0.84. The classifier also provides CNA plots and O6-methylguanine-DNA-methyltransferase (MGMT) promoter methylation status. The CNA plots were visually examined by a molecular biologist (L.C.M.) focusing on key genetic alterations in gliomas including 1p/19q codeletion, homozygous *CDKN2A/B* deletion, +7/−10 chromosomal gain/loss (one or both) and EGFR amplification.

Before proceeding with in silico analyses, the IDAT files were processed using RnBeads,^31^ and QC was performed. Subsequent analyses included the estimation of tumor purity (using the R-package InfiniumPurify^32^), identification of differentially methylated probes and regions (RnBeads-integrated combined rank analysis), cell-type deconvolution (using EpiDISH^33^), and estimation of stemness index (using the DMPsi signature^34^). Patients 1 and 6 did not pass QC. See Supplementary Methods for details.

### RNA Sequencing

RNA sequencing was performed at the Department of Genomic Medicine, Rigshospitalet, Copenhagen University Hospital, Denmark on paired tumors from 6 patients (information about which tumors were sequenced are given in Table 1). Larger areas of non-neoplastic tissue were macrodissected to increase tumor purity. Library preparation and indexing was performed using Illumina Stranded Total RNA Prep (cat.no: 20040529, Illumina) in conjunction with RNA UD Indexes Set A (cat.no: 20040553, Illumina). RNA sequencing was performed on a NovaSeq6000 (Illumina), with an SP flow cell (300 cycles).

For data analysis, we used the Nextflow^35^ nf-core/rnaseq (v.3.8.1) with GRCh38 as reference genome. QC filtering resulted in the exclusion of 2 samples: the true metastasis of patient 9 and the recurrent tumor of patient 17 (Supplementary Table 4). Expression-based subtyping was performed through the Single Sample Gene Set Enrichment Analysis (ssGSEA) with the ssgsea.GBM.classification R package^36^ (https://github.com/zhaoliang0302/ssgsea.GBM.classification). The gene set enrichment analysis was performed using the fgsea10 (v1.32.2) R package and the Molecular Signatures Database^37^ (MsigDB) hallmark gene sets.^38^ See Supplementary Methods for details.

### IHC and Automated Digital Quantitation

Automated quantitation of immune and stemness markers was performed using threshold-based classifiers (APPs) in the Visiopharm software (V2021.02.5.10297). Immune cells were identified based on the expression of CD8, CD68, and FOXP3, while stemness levels were identified based on the expression of SOX2, OLIG2, and neuronal marker MAP2 (Supplementary Table 5). The data output for each image analysis was either area fraction (positive area/μm^2^) or counts per area (cell count/μm^2^). See Supplementary Methods for details.

### Statistical Methods

Statistical calculations were performed using Graphpad Prism version 9.4.1 and 10.4.1, unless otherwise specified. Differences in mean between paired and unpaired data were tested using a 2-tailed paired and unpaired *t*-test, respectively. For binary variables, we used Fisher’s exact test to check for differences between groups. Analysis of correlation between tumor purity and calibrated class score was performed using Pearson’s correlation coefficient. Overall survival of glioblastoma patients was calculated starting from the date of histopathologic confirmed initial glioblastoma diagnosis to time of death. Kaplan–Meier survival curves were compared using a log-rank test. For all statistical tests, a significance level of 0.05 was chosen, and a Gaussian distribution of the test statistics was assumed.

### Ethics

This study was approved by the Regional Scientific Ethical Committee (project-ID: H-20027055) and the Danish Data Protection Agency (case number: P-2020-695) and was conducted in accordance with the Helsinki Declaration. None of the patients were registered in the Danish Tissue Application Register, and so the use of their tissue was not prohibited.

## Results

### Cohort Characteristics

**Figure 1.**
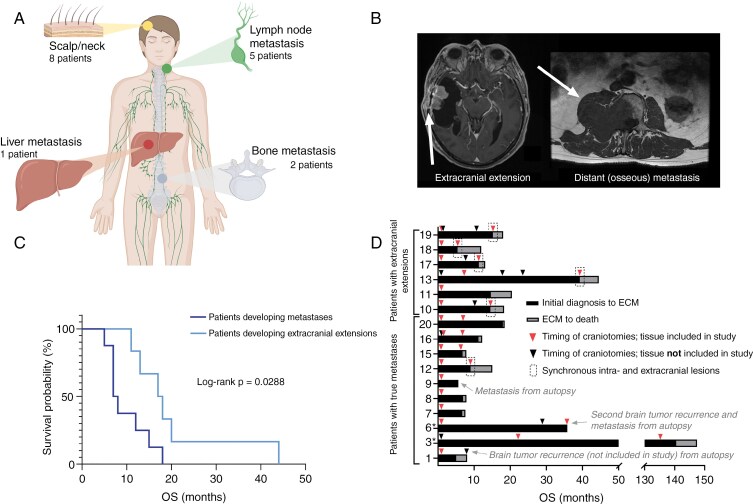
Illustrative overview of selected patient characteristics. (A) Overview of sites of metastasis. Created in BioRender. Kristensen, B. (2025) https://BioRender.com/esw3tdm. (B) MRI scans (*T*_1_-weighted postgadolinium axial) showing a contrast-enhancing tumor extending through a craniotomy burr hole with extracranial contrast-enhancing mass (arrow) (left) and distant osseous metastasis to the spine (arrow) with epidural compression (right) (*T*_1_-weighted axial without gadolinium). Created in BioRender. Kristensen, B. (2025) https://BioRender.com/fcogx5y. (C) Kaplan–Meier curves showing overall survival of glioblastoma patients with true metastasis (8 patients) and extracranial extension (6 patients) (log-rank *P *= .0288). Level of significance: *P* < .05. (D) Patient timeline from initial diagnosis (date of histopathologic report) to death including timepoints for craniotomies (date of histopathologic reports). *IDH-mutated tumors. *Abbreviations*: OS, Overall survival; ECM, Extracranial metastasis.

This cohort consisted of 14 patients with metastasis from glioblastoma, IDH-wildtype, WHO grade 4, and 2 patients with metastasis from lower-grade glioma. The number of patients complies with the expected number of cases within the current timeframe (1999-2019), as the reported frequency is 0.4%-0.5%.^5,6^ There are around 300 new cases of glioblastoma in Denmark per year,^39^ where approximately half of these are diagnosed at the Copenhagen University Hospital, Rigshospitalet. The 2 lower-grade gliomas were re-classified to comply with the WHO 2021 classification and comprised one astrocytoma, IDH-mutated, WHO grade 2 (progressing to WHO grade 3), and one oligodendroglioma, IDH-mutated and 1p/19q-codeleted, WHO grade 2 (progressing to WHO grade 3) (Table 1). The DKFZ brain tumor classifier supported the histopathological tumor diagnosis in most cases and a successful methylation class prediction (score ≥ 0.84) was achieved for 21 out of 31 tumors (68%) (Supplementary Table 6). One metastasis (patient 19) was originally diagnosed as a glioblastoma, IDH-wildtype, WHO grade 4 metastasis but was assigned to the methylation class malignant peripheral nerve sheath tumor (MPNST). This was supported by a patchy and focal S100 staining and a negative SOX10 staining, but with a morphology consistent with glioblastoma and similarities between the CNAs in the primary brain tumor and scalp lesion, the diagnosis was adjusted to glioblastoma, IDH-wildtype, WHO grade 4, with MPNST characteristics. Six glioblastomas displayed histological characteristics consistent with the gliosarcoma subtype in the primary or recurrent brain tumor. The extracranial metastases were distributed across the scalp/upper neck (8 patients), lymph nodes (5 patients), bone (2 patients), and liver (1 patient) (Figure 1A). Six of 8 patients with scalp/upper neck metastasis displayed contrast-enhancing continuous extracranial extensions of the brain tumors into the scalp. The remaining 10 patients were annotated as having true metastases due to a lack of connection between the primary brain tumor and metastasis (see Figure 1B for examples of the two types of spread).

The cohort consisted of 4 women and 12 men and median age at diagnosis was 56.5 years with no significant difference in age (*P *= .4375) between patients developing true metastasis and extracranial extension (Supplementary Table 7). We detected significantly more surgeries (craniotomies) in patients developing extracranial extensions (3 vs. 2, *P *= .0450); these patients also had a significantly higher overall survival (17.5 months vs. 7.5, *P = *.0288) (Figure 1C). Timelines for each patient are included in Figure 1D. Median overall survival among the glioblastoma patients (14 patients) was 12.5 months (range: 5-44 months) and median survival following detection of metastasis was 86 days (range: 15-200) (Supplementary Table 8). Overall survival in the lower-grade glioma patients was 147 and 35 months (patient 3 and 6, respectively) (Table 1).

The MGMT promoter was methylated in 2/14 glioblastoma patients (14%) and in both lower-grade glioma patients. For 11 patients, primary oncological treatment included radiation therapy and temozolomide, and at relapse, the patients received additional treatment as described in Table 1. Three of the glioblastoma patients were not treated upon first tumor recurrence, due to no signs of residual tumor (patients 13 and 17), and a poor condition post-surgery (patient 15).

### Radiological Cohort Characteristics

To investigate whether the location of brain tumors relative to certain anatomical regionscould impact the likelihood of metastasis, MRIs were thoroughly examined. We did not find any association between proximity of brain tumors to the dura, ventricles, or large vessels, and metastatic capability (Supplementary Table 9). However, we observed aberrant contrast-enhancement connecting with surgical defects in 5/6 patients with extracranial extensions, suggesting that tumor outgrowth occurred through burr holes made during primary surgical resection. For the sixth patient, we detected contrast-enhancements through the skull base, but whether outgrowth occurred through the craniotomy burr holes was not clear.

We further recorded radiological base characteristics for the primary brain tumors. Most tumors exhibited classical radiological characteristics of high-grade gliomas including a large contrast-enhancing part, with central necrosis, but most also had a non-enhancing tumor core.^40^ All contrast-enhancing tumors displayed large FLAIR hyperintensities surrounding the tumor core as seen in tumor edema. No tumors exhibited FLAIR/T2-mismatch; a sign attributed to IDH-mutant tumors. Total tumor volume spanned from 22.0 to 104.4 cm^3^. Overall, the primary brain tumors in our cohort exhibited similar radiological base characteristics regardless of the metastasis location, closely resembling glioblastomas that do not metastasize (Supplementary Table 10).

### Clonal Reconstruction Analysis Identifies the Primary Tumors as Origin of the Metastases

**Figure 2.**
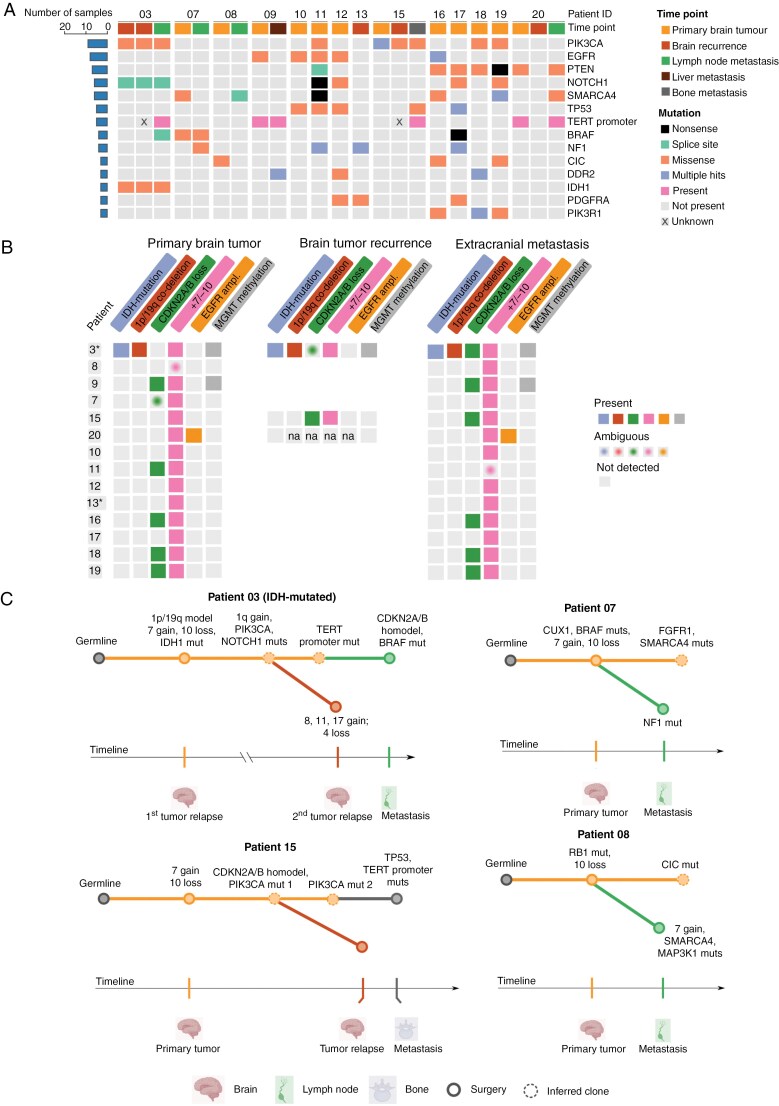
Molecular profiling of metastasizing adult gliomas. (A) Oncoprint showing the mutational landscape of sequential primary and recurrent gliomas, and extracranial metastases. Each column corresponds to individual samples systematically organized by patient, and clinically relevant mutated genes are listed along the *y*-axis. Different colors refer to clinical information and mutational functions. (B) Key molecular alterations relevant for glioma pathogenesis in sequential tumor samples, identified by visual inspection of CNA plots provided by the DKFZ brain tumor classifier V.12.5.^30^ Created in BioRender. Kristensen, B. (2025) https://BioRender.com/f7fj9z9. (C) Phylogenetic trees showing the clonal evolution of sequential tumors from 4 representative patients in the cohort with nodes representing clones and branches representing evolution paths. Branches are labeled with potential driver mutations and somatic copy number alterations (CNAs) inferred from the CNA plots obtained from the DKFZ brain tumor classifier V.12.5.^30^ Colored branches indicate divergent evolution, distinguishing each clone from the ancestral one, and the timeline is proportional to the number of days between surgeries. Inferred clonality is based on the variant allele frequency (VAF) of the mutations, with a lower VAF suggesting a subclonal variant. *Abbreviations*: *PIK3CA*, Phosphatidylinositol-4,5-bisphosphate 3-kinase catalytic subunit alpha; *EGFR*, Epidermal growth factor receptor; *PTEN*, Phosphatase and tensin homolog; *NOTCH1*, Notch receptor 1; *SMARCA4*, SWI/SNF related, matrix associated, actin dependent regulator of chromatin, subfamily a, member 4; TP53, Tumor protein p53; TERT, Telomerase reverse transcriptase; BRAF, B-Raf proto-oncogene, serine/threonine kinase; NF1, Neurofibromin 1; CIC, Capicua transcriptional repressor; DDR2, Discoidin domain receptor tyrosine kinase 2; *IDH1*, Isocitrate dehydrogenase 1; *PDGFRA*, Platelet derived growth factor receptor alpha; *PIK3R1*, Phosphoinositide-3-kinase regulatory subunit 1; *CDKN2A/B*, Cyclin dependent kinase inhibitor 2A/B; MGMT, O6-methylguanine-DNA-methyltransferase; Codel, Co-deletion; Mut, mutated, Muts, mutations; Homodel, Homozygous deletion; CUX1, Cut-like homeobox 1; FGFR1, Fibroblast growth factor receptor 1; RB1, RB transcriptional corepressor 1; MAP3K1, Mitogen-activated protein kinase 1. NA, not available due to a flat CNAs profile, meaning that CNAs could not be interpreted. *The first brain tumor recurrence was used instead of the primary brain tumor.

To explore the genetic landscape in metastasizing gliomas and further verify the glioma origin, sequencing-based mutational analysis was performed. A total of 1,491 non-synonymous somatic mutations from 413 genes were detected. The top 5 most recurrently altered genes harboring driver mutations were *PIK3CA*, *EGFR*, *PTEN*, *NOTCH1*, and *SMARCA4* (Figure 2A). We found no driver mutations in DNA Damage Repair (DDR) pathways such as mismatch-repair (MMR, including *MSH2*, *MSH6*, *MLH1*, *PMS2*) or evidence of microsatellite instability (MSIsensor2), nor homologous recombination (HR) (including *BRCA1*, *BRCA2*, *PALB2*). We found a non-significant trend toward elevated TMB in primary tumors with extracranial metastasis compared to primary tumors with distant metastasis (average 408 and 150, respectively, *P* = .05). In summary, we found no compelling evidence for DDR-mediated genomic instability as a prominent mechanism of metastasis in the cohort.

Four patients harbored hotspot alterations affecting the TERT promoter. Considering key CNAs (*CDKN2A/B* loss, +7/−10 gain/loss, EGFR amplification) and MGMT promoter methylation,^41^ 8/13 glioblastomas preserved their genetic profiles entirely during tumor progression (patients 9, 10, 12, 13, 16-19) (Figure 2B, Supplementary Table 11). MGMT promoter methylation status was retained throughout the disease course in all patients.

Next, we aimed to explore the evolutionary mechanisms driving tumor progression and metastasis. We evaluated the VAF (targeted DNA sequencing) and copy number (methylation-based) dynamics among longitudinally harvested tumors from 6 patients. Both the intracranial recurrences and extracranial metastases in 5/6 patients were found to originate and evolve from the primary tumors (as we did not have access to the primary tumor from patient 3, it was not possible to draw the same conclusion from this patient). A comparison of mutational profiles across matched primary, recurrent, and metastatic samples demonstrated that only in patients 15 and 20, the intracranial recurrences lacked mutations in oncogenic genes (Figure 2C, Supplementary Figure 3). Overall, these findings demonstrate that the glioma metastases originated from the primary tumors , suggesting that de novo tumors driving glioma metastases are rare.

### Methylation Class Shift Observed During Progression to Metastasis

To investigate epigenetic changes upon tumor progression, we performed methylation profiling on sequential tumors and applied the DKFZ brain tumor classifier. In total, 21/31 tumors (68%) were confidently assigned to a methylation class and received a class prediction score ≥ 0.84. Eight tumors (26%) received a score between 0.3 and 0.83 and 2 tumors received a score < 0.3 (6%) (Supplementary Figure 4A). The most common methylation class among primary glioblastomas was glioblastoma, IDH-wildtype, mesenchymal type (6/14, 43%) followed by glioblastoma, IDH-wildtype, RTK2 type (3/14, 21%). Similar distributions of the same methylation classes were found in the glioblastoma metastases (6/14, 43% and 2/14, 14%, respectively) (Supplementary Table 6).

We investigated whether tumor purity impacts methylation class score. Average tumor purity ranged from 15% to 92% with no significant difference between primary tumors, intracranial recurrences, and metastases (Supplementary Figure 4B). Methylation class scores correlated significantly with tumor purity (*R* = 0.3564, *P* = .0490) (Supplementary Figure 4C).

**Figure 3.**
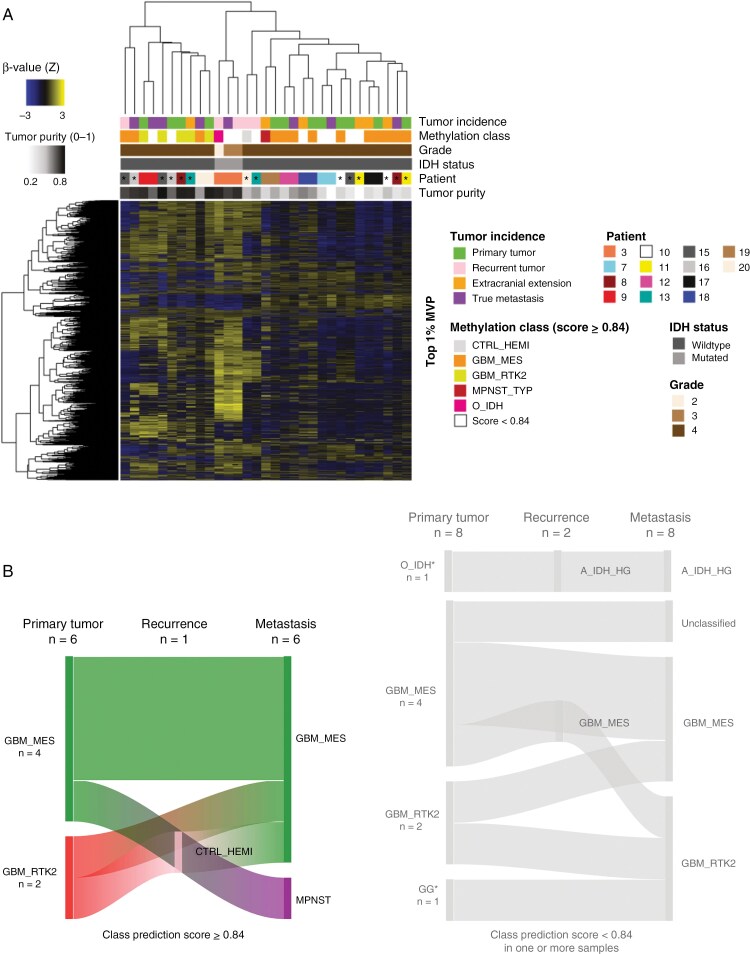
Genome-wide DNA methylation profiling (EPIC arrays) of sequential primary brain tumors, intracranial recurrences, and metastases. (A) Heatmap showing unsupervised hierarchical clustering of the top 1% most variable probes from methylation signatures. *Samples not clustering per patient. (B) Sankey diagram of predicted methylation classes for primary brain tumors, intracranial recurrences, and extracranial metastases as depicted by the DKFZ brain tumor classifier V.12.5.^30^ *The tumors are first intracranial recurrences. *Abbreviations:* IDH, Isocitrate dehydrogenase; MVP, Most variable probes.

Next, we performed unsupervised hierarchical clustering of the top 1% most variable probes across all samples. This revealed that the methylation profiles were predominantly patient-specific, indicating an overall stable methylation signature during tumor progression (8/14 patients) (Figure 3A). However, sequential samples from 6 patients (patients 8, 10, 11, 13, 15, 16, marked with asterisks) did not cluster together, suggesting less stable methylation signatures. According to the DKFZ brain tumor classifier, 2 of these tumors (patients 8 and 15) underwent methylation class shift during tumor progression when applying the ≥0.84 cutoff, and even more tumors underwent methylation class shift upon applying a less stringent cutoff (<0.84) (Figure 3B).

In summary, our findings highlight the potential involvement of epigenetic mechanisms in metastatic progression of glioblastomas.

### Global DNA Methylation Loss Occurs During Tumor Progression

To further investigate the role of epigenetics in glioma metastasis, we performed differential methylation analysis within and between groups. In total, 60,724 differentially methylated positions were identified between the primary tumors and metastases when combining patients developing true metastases and extracranial extensions. Most of these positions were hypomethylated in the metastases. The same pattern of hypomethylation upon tumor progression was observed when comparing sequential samples from patients developing extracranial extensions. Contrary, we detected zero differentially methylated positions when comparing primary tumors and true metastases in the group of patients developing true metastasis only, suggesting limitations due to low sample size. For all comparisons, there were too few differentially methylated regions to perform pathway analyses. Overall, we demonstrate that global DNA methylation loss occurs during tumor progression in our cohort.

### RNA Sequencing Reveals Subtype Switching During Tumor Progression

**Figure 4.**
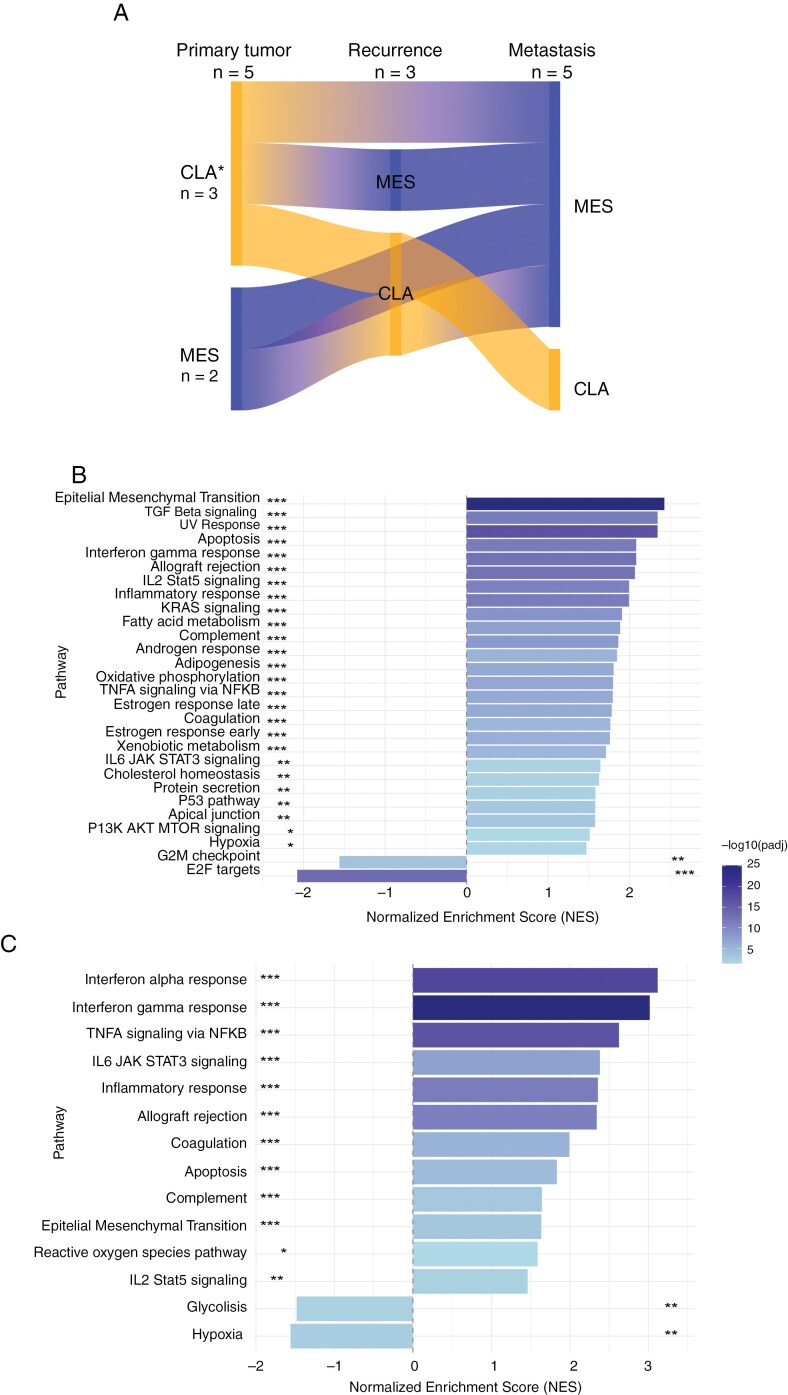
RNA sequencing on sequential tumor samples from 5 glioblastoma patients. (A) Sankey diagram of predicted transcriptional subtypes during tumor progression obtained by ssGSEA. (B) Pathway enrichment analysis comparing the primary tumors of patients developing true metastases with primary tumors of patients developing extracranial extensions. (C) Pathway enrichment analysis comparing intracranial recurrences and metastases from patients developing true metastases with intracranial recurrences and metastases from patients developing extracranial extensions. *For one of these tumors, the first brain tumor recurrence was used instead of the primary brain tumor; specifically the one retaining a classical profile throughout all 3 samples. *Abbreviations:* CLA, Classical; MES, Mesenchymal.

We performed ssGSEA on RNA sequencing data from 5 glioblastoma patients with extracranial extension and true metastasis to investigate potential changes in transcriptional subtypes during disease progression. Among 2 primary tumors assigned to the Classical subtype, both transitioned from Classical to Mesenchymal in the recurrence (if any) and metastasis. For one patient, we did not have access to the primary tumor and used the first recurrence instead. This tumor retained its Classical profile throughout disease progression. Conversely, among2 primary tumors identified as Mesenchymal, one retained its Mesenchymal profile in the metastasis (no recurrent tumor), while the other switched to Classical in the recurrence and back to Mesenchymal in the metastasis (Figure 4A).

To investigate whether true metastases and extracranial extensions develop from activation of different transcriptional programs, we performed ssGSEA comparing 1) primary tumors of patients with true metastasis versus primary tumors of patients with extracranial extension (Figure 4B), and 2) recurrences and metastases of patients with true metastasis versus recurrences and metastases of patients with extracranial extension (Figure 4C). In both comparisons, we found that patients with true metastases exhibited an increased activation of the epithelial-to-mesenchymal transition (EMT) pathway, in addition to an increase in various inflammatory response pathways such as TGF beta signaling, interferon gamma response, and IL2 STAT5 signaling.

In summary, we find evidence of transcriptional plasticity and RNA subtype switching during progression to metastasis.

### Stable Stemness Levels Observed During Tumor Progression to Metastasis

To study how stemness might affect tumor progression in glioma metastases, stemness levels in sequential samples were estimated using methylation data and stemness-related IHC markers. Neither methylation-based estimates of stemness nor IHC evaluation of stemness markers OLIG2, SOX2, and neuronal marker MAP2 indicated any difference in stemness during progression to metastasis (Supplementary Figures 5 and 6). These findings suggest that the stemness of tumors is not a key driver of metastatic progression from glioblastomas.

### The Immune Microenvironment in the Metastases Mirrors the Primary Brain Tumors With Only Few Exceptions

**Figure 5.**
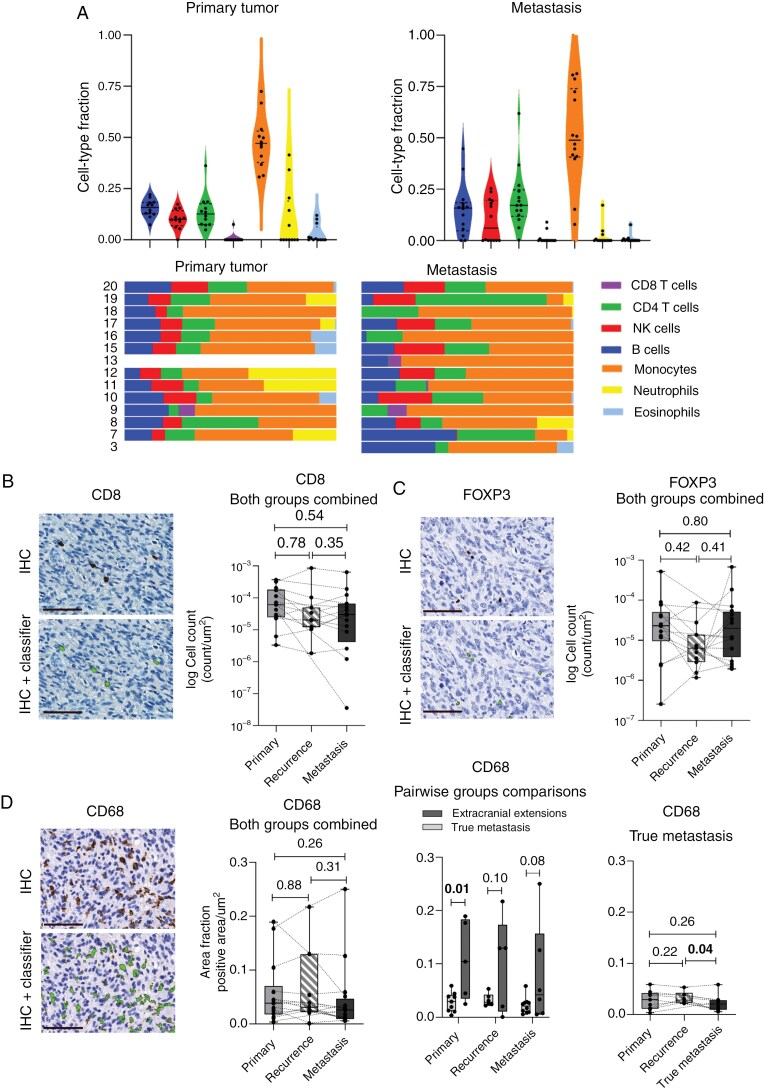
Profiling of the immune microenvironment in sequential primary brain tumors, intracranial recurrences, and extracranial metastases. (A) Violin and proportion plots showing cell-type deconvolution fractions of B cells, NK cells, CD4 T cells, CD8 T cells, monocytic-derived cells, neutrophils, and eosinophils in primary tumors and metastases. Median is shown as a horizontal line and 25^th^ and 75^th^ percentiles as dotted horizontal lines. Cell-type deconvolution data were not available from the primary tumor of patient 3 and 13, shown as blank bars in the proportion plots. (B) Quantitation of CD8^+^ T cells. (C) Quantitation of FOXP3^+^ Tregs. (D) Quantitation of TAMs marker CD68 shows a significantly higher expression in the primary brain tumors of patients developing extracranial extensions compared to primary brain tumors of patients developing true metastases (*P* = .01) (middle graph). There is a significantly lower expression in metastases compared to intracranial recurrences in patients developing true metastases (*P *= .04) (right graph). Level of significance: *P* < .05. Scale bar = 80 μm. *Abbreviations:* NK, Natural killer; CD4, Cluster of differentiation 4; CD8, Cluster of differentiation 8; IHC, Immunohistochemistry; FOXP3, Forkhead box P3; CD68, Cluster of differentiation 68. Created in BioRender. Kristensen, B. (2025) https://BioRender.com/i08z949.

The TME is known to be involved in the development of metastasis,^24^ but its role in glioma metastasis is unclear. To investigate this, we performed cell-type deconvolution (using methylation data) and quantitation of IHC TME markers on sequential samples in the cohort. Cell-type deconvolution revealed a dominance of monocytic-derived cells in the primary tumors and metastases. The levels of NK cells, CD4^+^ T cells, CD8^+^ T cells, monocytic-derived cells, neutrophils, and eosinophils were similar across sequential tumor samples with no significant difference between groups (Figure 5A, Supplementary Figure 7). We found B cells to be significantly less abundant in recurrent tumors compared to primary tumors (*P* = .03), which was also the case when performing subanalysis on sequential samples in patients developing true metastases only (*P* = .03; the same samples drove these results in both analyses). In the same subanalysis, we further observed a significant increase in B cells in the true metastases compared to recurrent tumors (*P* = .04). Importantly, these analyses are based on a limited sample size.

We next investigated the immune cell composition by quantitative estimates of immune cell markers and found that the amount of CD8^+^ T cells, FOXP3^+^ Tregs, and CD68^+^ macrophages did not change significantly between primary tumors, recurrences, and metastases, upon combining both types of spread (Figure 5B-D). Subanalyses revealed a significantly lower level of CD68^+^ macrophages in primary tumors from patients developing true metastases compared to primary tumors from patients developing extracranial extensions (*P =* .01, Figure 5D, middle graph). We also observed a slight decrease in CD68^+^ macrophages in metastases compared to intracranial recurrences among patients developing true metastases (*P =* .04, Figure 5D, right graph**).** The remaining subanalyses revealed no significant difference in immune cell levels between or within groups (Supplementary Figure 6).

To summarize, both methylation-based cell-type deconvolution and quantitation of IHC TME markers revealed overall similar immune cell compositions in primary brain tumors and extracranial metastases.

### Reduced GFAP Expression Observed in the Metastases

Next, we asked whether tumor progression and extracranial environments surrounding glioma metastases affect GFAP expression. There was a clear trend toward decreased or complete loss of GFAP expression in the metastases compared to the primary brain tumors. This pattern was also observed in the 2 lower-grade glioma patients (patients 3 and 6) (Supplementary Figure 8). This points towards loss of astrocyte-like differentiation upon tumor progression.

## Discussion

This study aimed to obtain deeper clinical, radiological, and molecular insights into metastasizing gliomas. Our cohort is the largest reported of its kind, with consecutive tumor samples from a total of 14 glioblastoma patients and 2 lower-grade glioma patients. Our extensive molecular analyses included mutational analysis, genome-wide DNA methylation profiling, RNA sequencing, and IHC.

We showed that adult gliomas can progress to true metastases and extracranial extensions. To our knowledge, no one has distinguished between extracranial extension and true metastasis in glioma patients before, nor have they investigated if survival is different between the two. Patients with extracranial extension had an increased survival compared to patients with true metastasis. This may be explained by increased survival leading to an increased risk of recurrences, which then leads to more craniotomies and thus an increased risk of developing extracranial extensions. Notably, 2 patients with scalp lesions had no MRI-detectable connection to a synchronous brain tumor and were initially annotated by us as having developed true metastases. However, after additional radiological inspections, we found that the scalp lesions in both cases were near craniotomy burr holes. Thus, intraoperative seeding along the surgical approach is a plausible explanation, as previously suggested.^13^ It is also worth noting that several of the lymph node metastases were located in the neck region, and so tumor dissemination via the meningeal lymphatic vessels is a possible route of spread.

The MRI characteristics of metastasizing glioblastomas in this study demonstrated many hallmark features commonly associated with non-metastasizing glioblastomas, including contrast-enhancement with heterogeneous appearances, central necrosis, and significant mass effect.^42,43^ These imaging findings are consistent with the well-established imaging phenotype of glioblastomas, which often exhibit disrupted blood-brain barriers and necrotic cores surrounded by viable tumor tissue. The tumor volumes spanned over a wide range, aligning with the variability reported in the literature for non-metastasizing glioblastomas.^44,45^ This lack of significant volumetric difference indicates that tumor volume does not influence metastatic potential.

By performing mutational analysis on sequential tumor samples, we found in 5 patients that the primary brain tumors were the clonal origins of the metastases, and that the recurrences and metastases evolved in parallel. This has been reported before, but the number of documented cases is scarce.^5,17,46^ With our findings, there is now increased evidence for this proposed mechanism of tumor evolution.

Methylation profiling revealed that at least 2 glioblastomas underwent a shift in methylation class during progression to metastasis. It has previously been shown that glioblastomas may undergo methylation class shift upon recurrence^47^; however, the present study is the first to show that this may occur during metastatic progression. We further detected recurrent hypomethylation among differentially methylated regions between the primary tumors and metastases, pointing towards global DNA methylation loss during tumor dissemination. Our data are in agreement with previous studies reporting progressive global loss of DNA methylation accompanying disease progression in other cancers^48,49^; however, follow-up investigations are necessary to understand the role of this phenomenon in glioblastoma metastasis.

RNA sequencing revealed dynamic subtype switching upon tumor progression to metastasis. Despite sample size limitations, we observed a general trend towards a shift to the Mesenchymal subtype in metastases. Together with the observed activation of the EMT pathway in tumors progressing to true metastases relative to tumors progressing to extracranial extensions, this could suggest that the activation of EMT plays a significant role in the development of true metastases. The same analysis revealed an upregulation of various immune response pathways in patients developing true metastases, and although the limited sample size prohibits drawing any firm conclusions, this could imply that the immune microenvironment is more actively involved when glioblastomas disseminate to distant sites compared to extracranialextensions..

In our analysis of stemness, both methylation-based stemness index and quantitation of stemness markers OLIG2 and SOX2 showed no significant difference between primary tumors, intracranial recurrences, and metastases. We previously demonstrated an increased OLIG2 expression in glioma recurrences, and SOX2 was increased in early recurrences, supporting the role of tumor stem-like cells in glioma progression.^50^ In the same manner, an elevated methylation-based stemness index has been reported in glioma recurrences.^34^ Importantly, these studies focused on recurrences and not metastases. Our results propose that stemness is not a driving factor for tumor progression in metastasizing gliomas.

Until now, it has not been investigated whether the immune microenvironment in glioma metastases differs from the primary brain tumor. Cell-type deconvolution and quantitation of immune cell markers revealed surprisingly similar immune cell compositions across primary and secondary tumor sites. Hence, the widely accepted notion of the immune privileged brain does not seem to extend to metastasizing gliomas. We did, however, detect higher levels of CD68^+^ macrophages in the primary tumors of patients developing extracranial extensions compared to those developing true metastases. Knowing that macrophages promote tumor cell migration,^51^ it can be speculated whether they are involved in promoting extracranial extension.

Regarding deeper mechanistic insight, our findings point toward the involvement of EMT in glioma metastasis. We found decreased GFAP expression in multiple metastases, reflecting loss of astrocyte-like differentiation upon tumor progression. One case-study reported the same reduced GFAP expression in glioblastoma metastases from 2 patients, together with an increased vimentin expression.^52^ This may point toward a clonal selection of mesenchymal-like tumor cells in the metastases. Our methylation analysis demonstrated plasticity on the epigenetic level, revealing methylation class shift to the mesenchymal subtype upon metastatic progression. Indeed, the same pattern was observed on the transcriptional level. Our cohort further consisted of an unusual high number of gliosarcomas, and we speculate whether the mesenchymal component characteristic to this subtype increased the likelihood of metastasis.

In conclusion, we show that primary tumors are the clonal origin of glioma metastases. Extracranial extension of brain tumors into the scalp is distinct from true, distant metastases. We observed epigenetic and transcriptional subtype plasticity upon tumor progression, demonstrated by methylation class and transcriptional subtype switch. The immune cell composition of the TME is generally maintained regardless of the location of the metastasis, thereby suggesting that potentially future efficient TME-directed therapies targeting gliomas may also be efficient against glioma metastases.

## Supplementary Material

noaf178_Supplementary_Data

## Data Availability

Data will be made available upon reasonable request.
