# Supplementary material for: Clinical, radiological, and molecular insights into extracranial metastases from adult gliomas
Source: Neuro Oncol. 2025 Aug 16;28(1):99–114. doi: 10.1093/neuonc/noaf178 (PMC12962640; doi:10.1093/neuonc/noaf178)
Supplement: noaf178_Supplementary_Data [file noaf178_supplementary_data.zip › noaf178_suppl_Supplementary.docx]

**SUPPLEMENTARY METHODS**

**Radiological tumor characterization**

Brain MRIs of patients in the cohort were reviewed by a radiology resident (J.C.) together with a neuroradiologist (J.F.C.). Primary brain scans (MRI of the primary brain tumor) and secondary brain scans (MRI at the time of metastasis or extracranial extension) were thoroughly evaluated using contrast-enhanced T1, T2 and Fluid-Attenuated Inversion Recovery (FLAIR) sequences. To identify potential routes of tumor cell dissemination, brain tumor distance to the dura mater, large vessels, and ventricles were evaluated. The distance of the brain tumors to dura and ventricles were classified as either “in contact with” or within a distance of < 2 mm or > 2 mm to these structures. Brain tumor distance to large vessels was categorized as either “in contact with” a vessel or as “no contact”. Dural metastasis was evaluated as “proximal dural metastasis” if the metastasis was located within the craniotomy area, “distant metastasis” if it was located elsewhere, or as “no dural metastasis”. Primary and/or secondary brain scans were not available for five patients. In two of these cases, MRIs obtained four to five months prior to the detection of metastasis were evaluated instead.

Additionally, a number of radiological base characteristics were recorded for the primary brain tumors; contrast-enhancement pattern (rim, solid, or mixed), the presence of central necrosis, the presence of FLAIR/T2 mismatch, and the presence of mass effect exerted by the tumor. Volumetry was performed for the total tumor volume, the contrast-enhancing areas, the necrotic areas, and the surrounding FLAIR changes. Volumetry was performed manually using Slicer 3D^1,2^.

**Targeted DNA sequencing**

Targeted next-generation sequencing (NGS) was performed at the Department of Genomic Medicine, Rigshospitalet, Copenhagen University Hospital, Denmark (information about which tumors were sequenced are given in **Table 1**). DNA was quantified using Qubit (Thermo Fisher) and 106-300 ng was used as starting material. Targeted sequencing was performed using the Illumina TruSight Oncology 500 (TSO500) panel covering 523 cancer-associated genes. The protocol was performed according to the manufacturer’s instructions and paired-end sequencing was carried out on NovaSeq 6000 (Illumina).

**Pre-processing of DNA sequencing data**

The resulting FastQ files underwent quality control (QC) using “FastQC 0.11.8”^3^ “FastQC: A Quality Control Tool for High Throughput Sequence Data”. Available online at: http://www.bioinformatics.babraham.ac.uk/projects/fastqc/) and were aligned to the human reference genome (GRCh38) using “BWA MEM 0.7.15”^4^. Data pre-processing was performed following the GATK Best Practices^5^ using “GATK 4.1.9.0”: “MarkDuplicates” was used to identify PCR and optical duplicates; base quality score recalibration was performed using “BaseRecalibrator” followed by “ApplyBQSR”. Coverage statistics were gathered using “CollectWgsMetrics” and “mosdepth 0.3.1”^6^. QC of the final BAM files was performed by running “CollectAlignmentSummaryMetrics”, “CollectBaseDistributionByCycle”, “CollectGcBiasMetrics”, “CollectInsertSizeMetrics” and “QualityScoreDistribution”. QC results were inspected using “MultiQC 1.12”^7^. “Somalier 0.2.11”^8^, a genetic distance-based tool, was applied to verify the correct pairing of the samples. Patients 1 and 6 were excluded during QC. QC results are summarized in **Supplementary Table 2.**

**Variants calling and annotation**

Somatic single-nucleotide variants (SNVs) were called for each tumor sample using “MuTect2”^9^ (in accordance to the GATK Best practices, using “GATK 4.1.9.0”). “MuTect2” was run using an in-house panel of normals and the “gnomAD” (“af-only-gnomad.hg38.vcf.gz”) germline resource as additional controls, in order to minimize the potential for germline artifacts. Additionally, “MuTect2” was employed to jointly call SNVs across all the samples (multi-sample mode), for the patients for which more than 1 sample was available. SNVs’ QC was performed using “varlap 0.1.0.0” (https://github.com/bjpop/varlap) and functional annotation was performed using the “Ensembl Variant Effect Predictor 99.0”^10^.

To ensure accurate variant calling and minimize false-negative results, we implemented strict QC criteria across all variants analyzed. For each SNV (**Supplementary Table 3**), we computed read depth metrics using samtools mpileup^11^ and established reporting thresholds. Variants were classified as "present" when meeting our standard variant calling criteria (VAF > 5%; coverage for the alternative base > 5 reads; total coverage > 10 reads), "not present" when having sufficient coverage but no supporting reads for the alternate allele, and "unknown" when having insufficient coverage to make a confident call. This was particularly critical for challenging genomic regions such as the TERT promoter, which has high GC content. For TERT promoter mutations specifically, we therefore additionally required a sample to have tumor purity >10%.

Tumor mutational burden was computed as the ratio between non-synonymous mutations and the nucleotide size of the capture kit (TSO500) (**Supplementary Table 2**). Microsatellite instability was assessed using MSIsensor2 (https://github.com/niu-lab/msisensor2).

**Clonal evolution reconstruction**

We reconstructed the clonal evolution of tumors by analyzing differences in distribution of somatic variants across multiple samples from each patient. For each SNV, we computed the variant allele frequency (VAF), defined as the fraction of sequencing reads carrying a mutation, which is a readout of the proportion of DNA mutated in the sequenced tissue:

$$VAF=\frac{t\_alt\_counts}{(t\_alt\_counts + t\_ref\_counts)}$$

Where “t_alt_counts” represents the number of reads supporting the alternate allele and “t_ref_counts” the number of reads supporting the reference allele. We used the VAF of SNVs to infer the proportion of cells bearing the SNV (cellular prevalence, or CP). We then clustered SNVs with similar CPs (see **Supplementary Figure 2**), assuming that these occurred within a single distinct clone. Unlike approaches that rely on cancer cell fractions (CCFs) calculations, which require allele-specific copy number alterations (CNAs), our approach was adapted to work with the tumor-only VAF. Since DNA-based copy-number calls where not possible to extract from the FFPE-based panel sequencing data, we leveraged available methylation SNP array data to estimate copy-number states across the genome, to identify larger amplifications (50-100kb) and deletions at key glioblastoma-associated genes. We compared these SNP array profiles across different tumor samples from each patient to identify major copy number changes during tumor evolution, with particular attention to relevant driver alterations such as EGFR amplification.

We employed a binary classification approach for somatic mutations, as previously described^12,13^. Mutations were classified into three categories:

1. **Trunk mutations:** SNVs or CNAs present in all time-points from a patient with consistently high cellular prevalence for the SNVs (CP > 0.6, **Supplementary Figure 2**). These mutations likely occurred early in tumor evolution and are present in the most recent common ancestor of all sampled tumor cells.
2. **Branch mutations:** Subclonal (CP<=0.6) SNVs or CNAs present in two or more but not all tumor cell populations, suggesting they emerged after the initial clonal expansion but before the divergence of specific subclones.
3. **Leaf mutations:** Subclonal (CP<=0.6) SNVs or CNAs present in only a single tumor sample, representing private mutations that emerged late in the evolutionary history of that specific tumor.

The temporal ordering of mutations was inferred by combining information on:

1. The presence/absence pattern across samples
2. The cellular prevalence of each mutation
3. The chronological timing of surgical interventions when samples were obtained

Clustered mutations were ordered by their cellular prevalence across multiple samples from early to late, following principles established in previous studies^12,14,15^. The resulting phylogenetic trees were manually curated and constructed based on these principles.

**Genome-wide DNA methylation profiling**

Prior to DNA methylation profiling, the extracted DNA underwent bisulfite conversion using the EZ DNA MethylationTM Kit (Zymo Research). Degraded DNA was restored using the Infinium FFPE DNA Restore Kit (Illumina). Genome-wide DNA methylation analysis was carried out at Life & Brain GmbH, Platform Genomics in Bonn, Germany. DNA was hybridized to an array-based Illumina Infinium MethylationEPIC (850K) BeadChip and scanned using the Illumina iScan platform. Output data were provided as paired IDAT files which were uploaded to the online DNA methylation-based CNS tumor classifier created by the German Cancer Research Center (DKFZ) and Heidelberg University^16^. Using classifier version 12.5, we included all cases with a family- and class prediction score ≥ 0.84. Together with these scores, we retrieved we also obtained plots of CNAs and O6-methylguanine-DNA-methyltransferase (MGMT) promoter methylation status. The CNA plots were visually examined by a molecular biologist (L.C.M.) focusing on key genetic alterations in gliomas including 1p/19q codeletion, homozygous CDKN2A/B deletion, +7/-10 chromosomal gain/loss (one or both) and EGFR amplification.

Before proceeding with additional in silico analyses, the IDAT files were processed using RnBeads^17^. Poor quality probes (determined by the Greedycut algorithm), sex chromosome probes, and probe sequences overlapping with >2 common single nucleotide polymorphisms (SNPs) were removed. A total of 792.788 probes and 31 samples passed QC. Patients 1 and 6 did not pass QC, leaving a total of 14 patients included in these analyses (13 patients with glioblastoma, IDH-wildtype, WHO grade 4 and one patient with oligodendroglioma, IDH-mutated and 1p/19q co-deleted, WHO grade 2, progressing to grade 3). Data were subjected to beta-mixture inter-quartile (BMIQ) normalization. Methylation levels were quantified using β-values (0-1). Probes were annotated according to the EPIC hg38 manifest (Illumina). Differentially methylated probes and regions were identified using the RnBeads-integrated combined rank analysis, upon performing the following comparisons: i) all primary tumors versus all metastases (both groups combined), ii) Primary tumors versus true metastases (patients developing true metastases only), iii) Primary tumors versus extracranial extensions (patients developing extracranial extensions only), iv) Primary tumors versus recurrences (patients developing true metastases only), and v) Recurrences versus true metastases (patients developing true metastases only). Tumor purity was estimated using the R-package InfiniumPurify^18^, where both the glioblastoma multiforme and the lower-grade gliomas reference cohorts provided by The Cancer Genome Atlas (TCGA) were used^19,20^. Cell-type deconvolution was performed using EpiDISH^21^to predict relative cell-type fractions for B cells, NK cells, CD4+ T cells, CD8+ T cells, monocytes, neutrophils, and eosinophils. Stemness of samples was inferred using the DMPsi signature^22^. Methylation values for 92% (57/62) of the DMPsi signature probes were detected and the prespecified weighted linear formula was used to calculate an overall stemness index score for each sample. Stemness scores were subsequently Z-transformed.

**RNA sequencing**

Paired tumors from six patients (patient 8, 9, 12, 13, 17, and 19) were included for RNA sequencing. Depending on tissue age, 4-8 FFPE sections of 10 µm were used as starting material for RNA isolation. Tumors containing large areas of non-neoplastic tissue were subject to macrodissection to increase tumor purity, and paraffin was removed to increase RNA quality. RNA extraction was performed using the FormaPure RNA Kit (cat.no: C19157, Beckman Coulter) according to the manufacturer’s guidelines. RNA quality was assessed on a Bioanalyzer (Agilent Technologies) using the Agilent RNA 6000 Nano Kit (cat.no: 5067-1511, Agilent Technologies) and total RNA nanochip (DV_200_). Prior to library preparation, RNA concentration/purity was measured using Nanodrop (DeNovix Inc.). For library preparation and indexing, we used Illumina Stranded Total RNA Prep (cat.no: 20040529, Illumina) in conjunction with RNA UD Indexes Set A (cat.no: 20040553, Illumina). Library purification was performed using the AMPure XP beads (cat.no: A63881, Beckman Coulter) and RNA Clean XP (cat.no: A63987, Beckman Coulter). cDNA concentration was measured with the Qubit dsDNA Broad Range Assay Kit (cat.no: Q33266, Thermo Fisher), and final library quality was assessed on a TapeStation (Agilent Technologies) using the High Sensitivity D5000 ScreenTape assay (cat.no: 5067-5593, Agilent Technologies. RNA sequencing was performed on a NovaSeq6000 (Illumina), with an SP flow cell (300 cycles).

FASTQ files were processed using the Nextflow^23^ nf-core^24^/rnaseq pipeline (v.3.8.1). For the alignment and quantification, STAR^25^ and Salmon^26^ were used, respectively, with Singularity (v3.6.2) as the profile option and GRCh38 as the reference genome. QC filtering resulted in the exclusion of two samples, specifically the true metastasis of patient 9 and the recurrent tumor of patient 17, due to low read depth, with an average of 3 million mapped reads versus the cohort average of 56 million. Batch inspection was performed using DESeq2^27^. Somalier^8^ (v0.2.11), a tool based on genetic distance, was used to verify the correct pairing of samples. Stromal cell contamination was estimated using PUREE^28^, resulting in an average purity of 75% across all the samples.

Expression-based subtyping was performed through the Single Sample Gene Set Enrichment Analysis (ssGSEA), specifically with the ssgsea.GBM.classification R package^29^ (https://github.com/zhaoliang0302/ssgsea.GBM.classification). The gene set enrichment analysis (GSEA) was performed using the fgsea10 (v1.32.2) R package and the Molecular Signatures Database^30^ (MSigDB) hallmark gene sets^31^. The GSEA was based on the top ranked differentially expressed (DE) genes, resulting from DESeq2^27^ DE analysis comparing: i) the primary tumors of patients with true metastasis versus patients with extracranial extensions and ii) the later time-points of patients with true metastasis versus patients with extracranial extensions.

**IHC and automated digital quantitation**

Immune cells were identified based on the expression of CD8, CD68 and FOXP3, while stemness levels were identified based on the expression of SOX2, OLIG2, and neuronal marker MAP2. FFPE tumor tissue was cut in 3 μm sections and stained manually with hematoxylin and eosin (HE). IHC was performed using the Benchmark Discovery Ultra platform (Ventana Medical Systems Inc) and Dako Omnis (Agilent Technologies). The slides were digitalized at 40x using the NanoZoomer Oncotopics® Scan by Visiopharm (Hamamatsu Photonics) and imported into the image analysis module of the Visiopharm software (V2021.02.5.10297). Regions of interest (ROIs) containing vital tumor areas were manually outlined and areas with large necrosis, hemorrhage, vessels, blurring, or folded tissue were excluded. Using a pixel-based algorithm, threshold-based classifiers (APPs) were programmed in the Visiopharm software to detect each individual marker, enabling automated quantitation of marker expression. The data output for each image analysis was area fraction (positive area/μm^2^) for SOX2, OLIG2, CD68 and MAP2, and counts per area (cell count/μm^2^) for CD8 and FOXP3.

**REFERENCES**

1. 3D Slicer image computing platform.https://www.slicer.org/. Accessed January 21, 2025.

2. Fedorov A, Beichel R, Kalpathy-Cramer J, et al. 3D Slicer as an image computing platform for the Quantitative Imaging Network. Magn Reson Imaging. 2012;30(9):1323-1341.

3. Andrews S. FastQC: a quality control tool for high throughput sequence data.https://www.bioinformatics.babraham.ac.uk/projects/fastqc/. 2010.

4. Li H, Durbin R. Fast and accurate short read alignment with Burrows-Wheeler transform. Bioinformatics. 2009;25(14):1754-1760.

5. Van der Auwera GA & O’Connor BD. *Genomics in the Cloud: Using Docker, GATK, and WDL in Terra*. 1st ed. O’Reilly Media; 2020. O’Reilly Media

6. Pedersen BS, Quinlan AR. Mosdepth: quick coverage calculation for genomes and exomes. Bioinformatics. 2018;34(5):867-868.

7. Ewels P, Magnusson M, Lundin S, et al. MultiQC: summarize analysis results for multiple tools and samples in a single report. Bioinformatics. 2016;32(19):3047-3048.

8. Pedersen BS, Bhetariya PJ, Brown J, et al. Somalier: rapid relatedness estimation for cancer and germline studies using efficient genome sketches. Genome Med. 2020;12(1):62.

9. Benjamin D, Sato T, Cibulskis K, et al. Calling Somatic SNVs and Indels with Mutect2. bioRxiv. Published online January 1, 2019:861054.

10. McLaren W, Gil L, Hunt SE, et al. The Ensembl Variant Effect Predictor. Genome Biol. 2016;17(1):122.

11. Danecek P, Bonfield JK, Liddle J, et al. Twelve years of SAMtools and BCFtools. Gigascience. 2021;10(2).

12. Jamal-Hanjani M, Wilson GA, McGranahan N, et al. Tracking the Evolution of Non-Small-Cell Lung Cancer. N Engl J Med. 2017;376(22):2109-2121.

13. McGranahan N, Favero F, de Bruin EC, et al. Clonal status of actionable driver events and the timing of mutational processes in cancer evolution. Sci Transl Med. 2015;7(283):283ra54.

14. Gerlinger M, Rowan AJ, Horswell S, et al. Intratumor heterogeneity and branched evolution revealed by multiregion sequencing. N Engl J Med. 2012;366(10):883-892.

15. Dentro SC, Wedge DC, Van Loo P. Principles of Reconstructing the Subclonal Architecture of Cancers. Cold Spring Harb Perspect Med. 2017;7(8).

16. Capper D, Stichel D, Sahm F, et al. Practical implementation of DNA methylation and copy-number-based CNS tumor diagnostics: the Heidelberg experience. Acta Neuropathol. 2018;136(2):181-210.

17. Assenov Y, Müller F, Lutsik P, et al. Comprehensive analysis of DNA methylation data with RnBeads. Nat Methods. 2014;11(11):1138-1140.

18. Qin Y, Feng H, Chen M, et al. InfiniumPurify: An R package for estimating and accounting for tumor purity in cancer methylation research. Genes Dis. 2018;5(1):43-45.

19. Brennan CW, Verhaak RGW, McKenna A, et al. The somatic genomic landscape of glioblastoma. Cell. 2013;155(2):462.

20. Brat DJ, Verhaak RGW, Aldape KD, et al. Comprehensive, Integrative Genomic Analysis of Diffuse Lower-Grade Gliomas. N Engl J Med. 2015;372(26):2481-2498.

21. Teschendorff AE, Breeze CE, Zheng SC, et al. A comparison of reference-based algorithms for correcting cell-type heterogeneity in Epigenome-Wide Association Studies. BMC Bioinformatics. 2017;18(1):105.

22. Malta TM, Sokolov A, Gentles AJ, et al. Machine Learning Identifies Stemness Features Associated with Oncogenic Dedifferentiation. Cell. 2018;173(2):338-354.e15.

23. Di Tommaso P, Chatzou M, Floden EW, et al. Nextflow enables reproducible computational workflows. Nat Biotechnol. 2017;35(4):316-319.

24. Ewels PA, Peltzer A, Fillinger S, et al. The nf-core framework for community-curated bioinformatics pipelines. Nat Biotechnol. 2020;38(3):276-278.

25. Dobin A, Davis CA, Schlesinger F, et al. STAR: ultrafast universal RNA-seq aligner. Bioinformatics. 2013;29(1):15-21.

26. Patro R, Duggal G, Love MI, et al. Salmon provides fast and bias-aware quantification of transcript expression. Nat Methods. 2017;14(4):417-419.

27. Love MI, Huber W, Anders S. Moderated estimation of fold change and dispersion for RNA-seq data with DESeq2. Genome Biol. 2014;15(12):550.

28. Revkov E, Kulshrestha T, Sung KWK, et al. PUREE: accurate pan-cancer tumor purity estimation from gene expression data. Commun Biol. 2023;6(1):394.

29. Wang Q, Hu B, Hu X, et al. Tumor Evolution of Glioma-Intrinsic Gene Expression Subtypes Associates with Immunological Changes in the Microenvironment. Cancer Cell. 2017;32(1):42-56.e6.

30. Subramanian A, Tamayo P, Mootha VK, et al. Gene set enrichment analysis: a knowledge-based approach for interpreting genome-wide expression profiles. Proc Natl Acad Sci U S A. 2005;102(43):15545-15550.

31. Liberzon A, Birger C, Thorvaldsdóttir H, et al. The Molecular Signatures Database (MSigDB) hallmark gene set collection. Cell Syst. 2015;1(6):417-425.
