# Supplementary material for: Clinical, radiological, and molecular insights into extracranial metastases from adult gliomas
Source: Neuro Oncol. 2025 Aug 16;28(1):99–114. doi: 10.1093/neuonc/noaf178 (PMC12962640; doi:10.1093/neuonc/noaf178)
Supplement: noaf178_Supplementary_Data [file noaf178_supplementary_data.zip › noaf178_suppl_Supplementary_Tables_1-11_Figures_1-8.docx]

**SUPPLEMENTARY TABLES AND FIGURE LEGENDS**

**Supplementary Table 1.** Overview of patients with other suspected metastases detected by imaging

| **Patient 3** |
| --- |
| 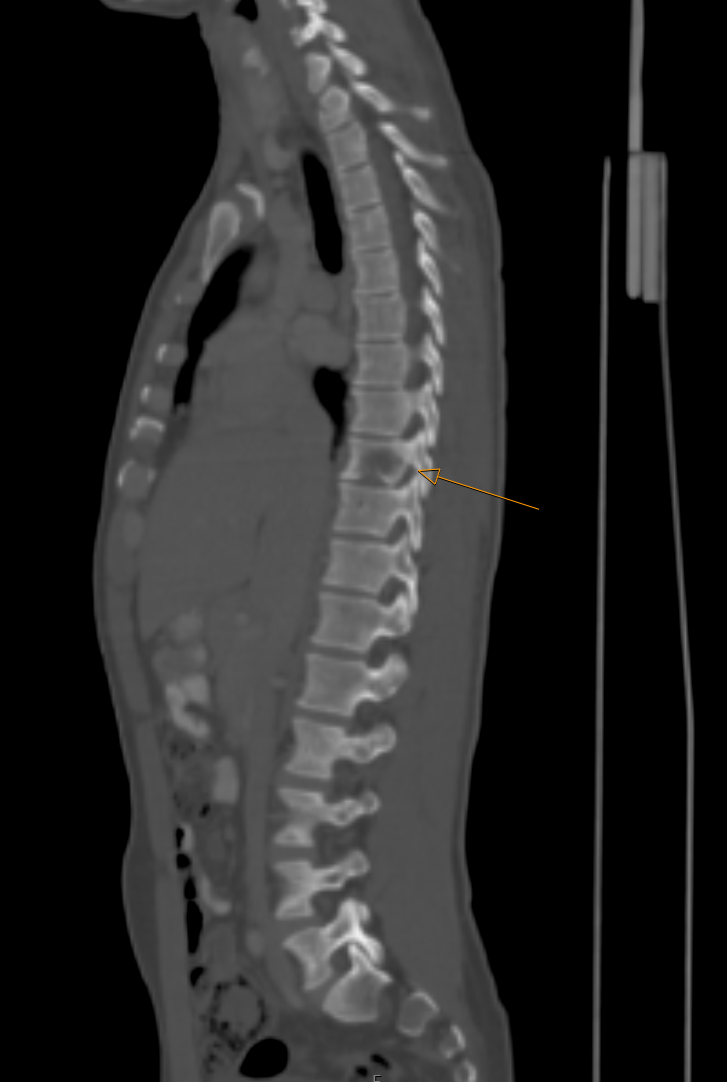  Parasagittal contrast-enhanced CT of the thorax and abdomen showing an osteolytic tumor in the vertebral body of Th9 (orange arrow). The tumor was F-18-FDG-PET positive and suspect of metastasis. |
| **Patient 8** |
| No scans available. Suspected metastases to lymph nodes in the neck area and mediastinum, as well as suspected metastases to liver, bone, and lungs. This patient appears as case 1 in a published case study^1^. |
| **Patient 15** |
| 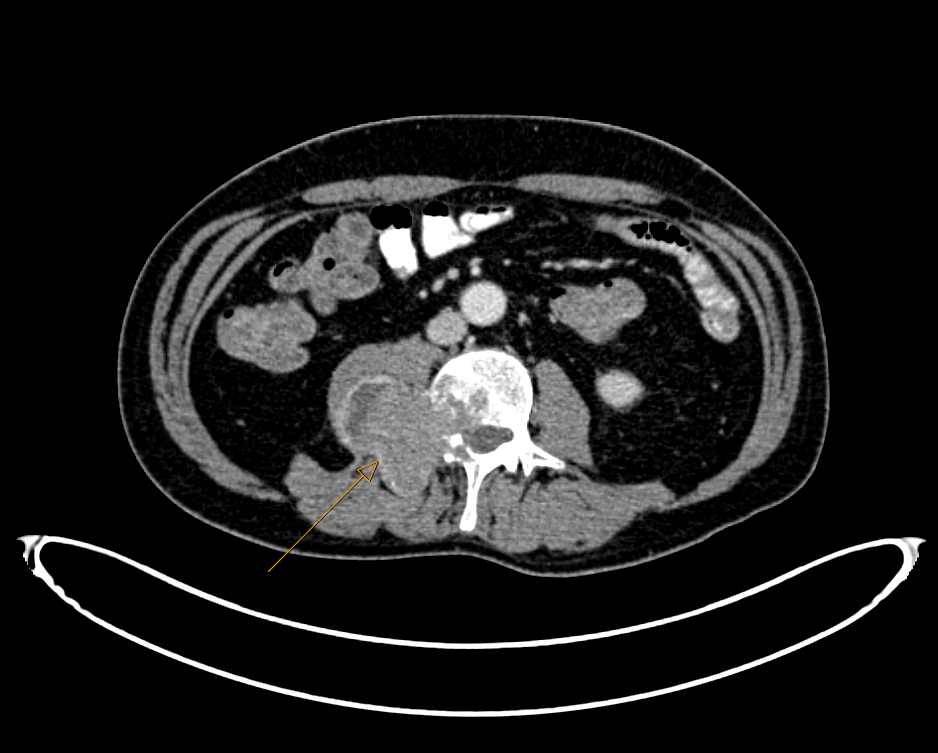  Axial contrast enhanced CT at the L3 level showing a large soft tissue mass in the body, the right pedicle, and the right upper articular process of the L3, invading the nearby paravertebral muscle (orange arrow).  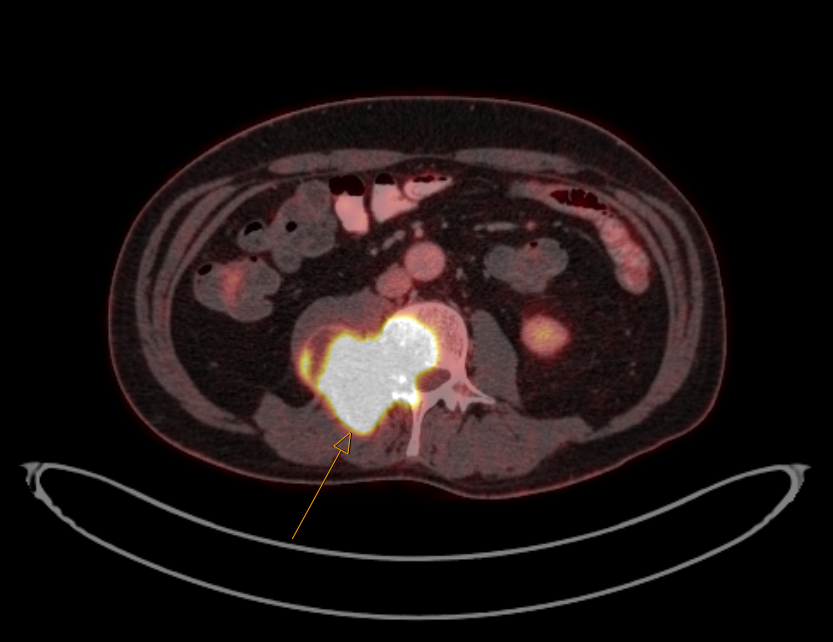  Axial F-18-FDG PET fused to contrast enhanced CT (including peroral contrast) showing increased FDG-uptake a large soft tissue mass in the body, the right pedicle, and the right upper articular process of the L3, invading the nearby paravertebral muscle (orange arrow).  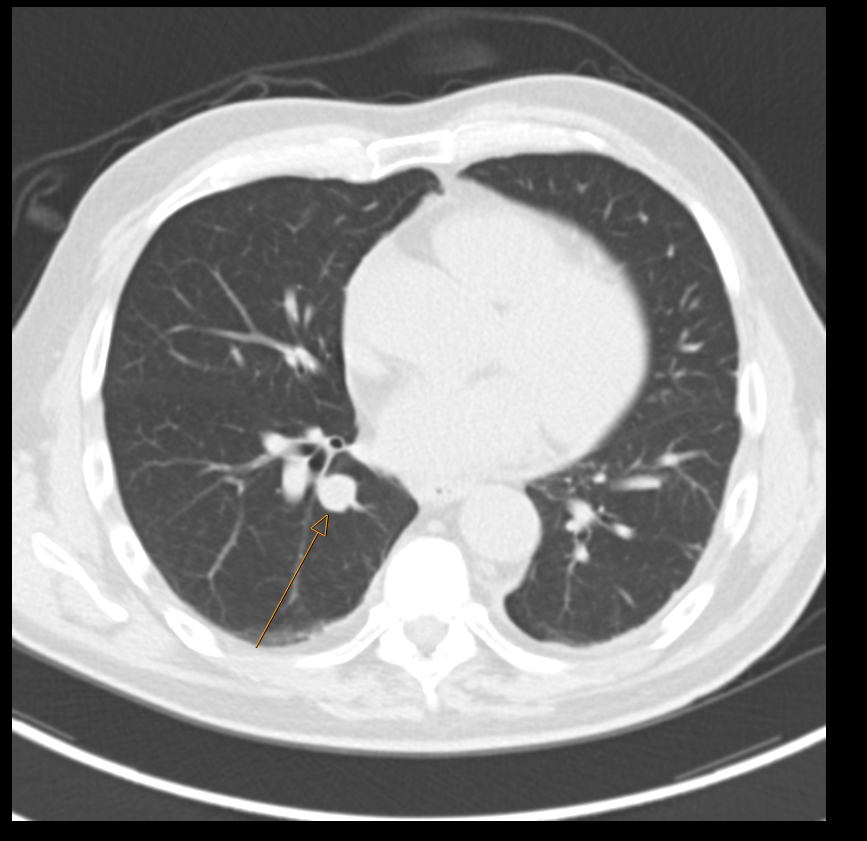  Axial non-contrast enhanced CT under breath hold showing a 15 mm nodule in the right lower lobe suspect of metastasis.  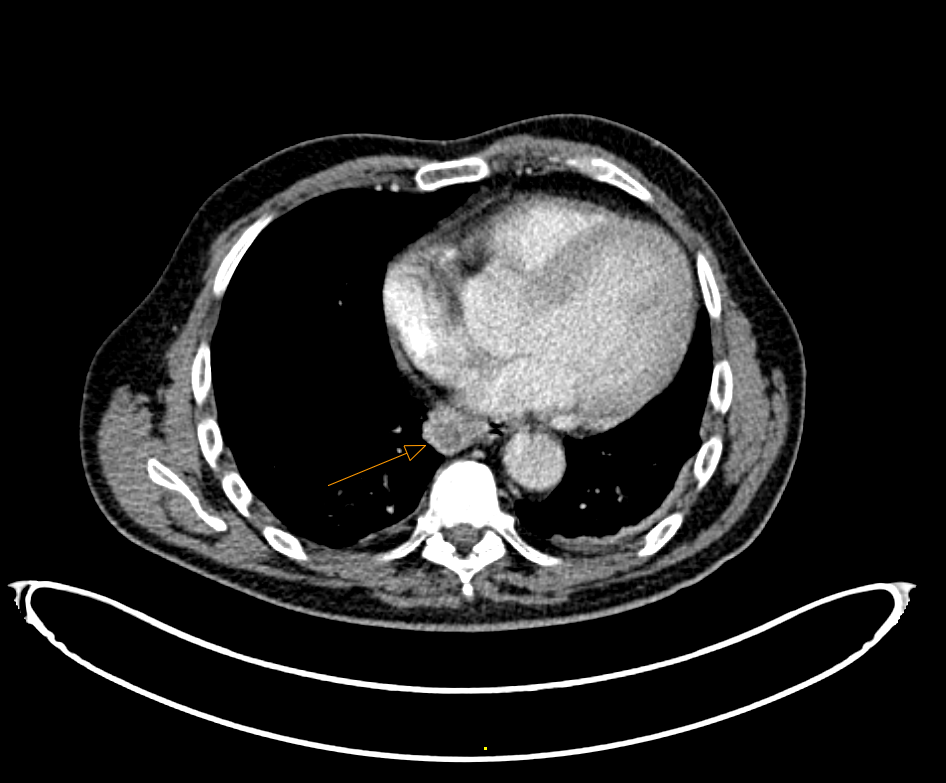  Axial contrast enhanced CT of the thorax showing an enlarged lymph node in the right mediastinum with peripheral enhancement and a necrotic center suspect of metastasis.  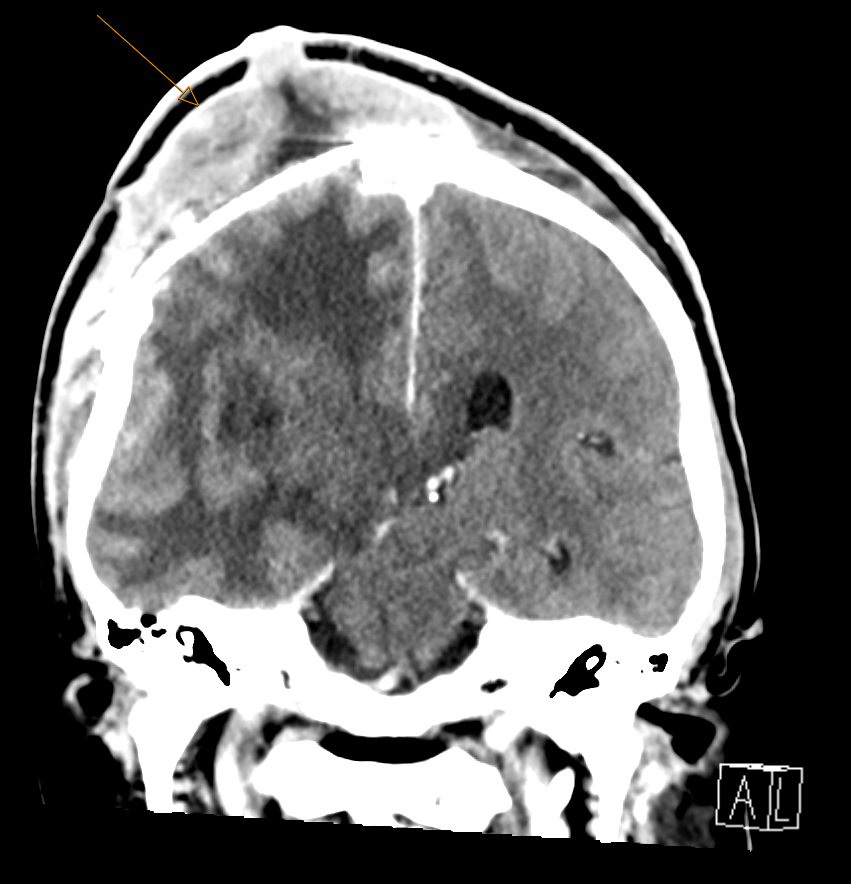  Coronal contrast enhanced CT of the skull post craniectomy showing a large subcutaneous mass overlying the craniectomy site (orange arrow) with tumor extension to the skin, the brain parenchyma and most likely the bone. There is underlying perifocal edema and blood in the subarachnoid space with considerable mass effect. |
| **Patient 20** |
| 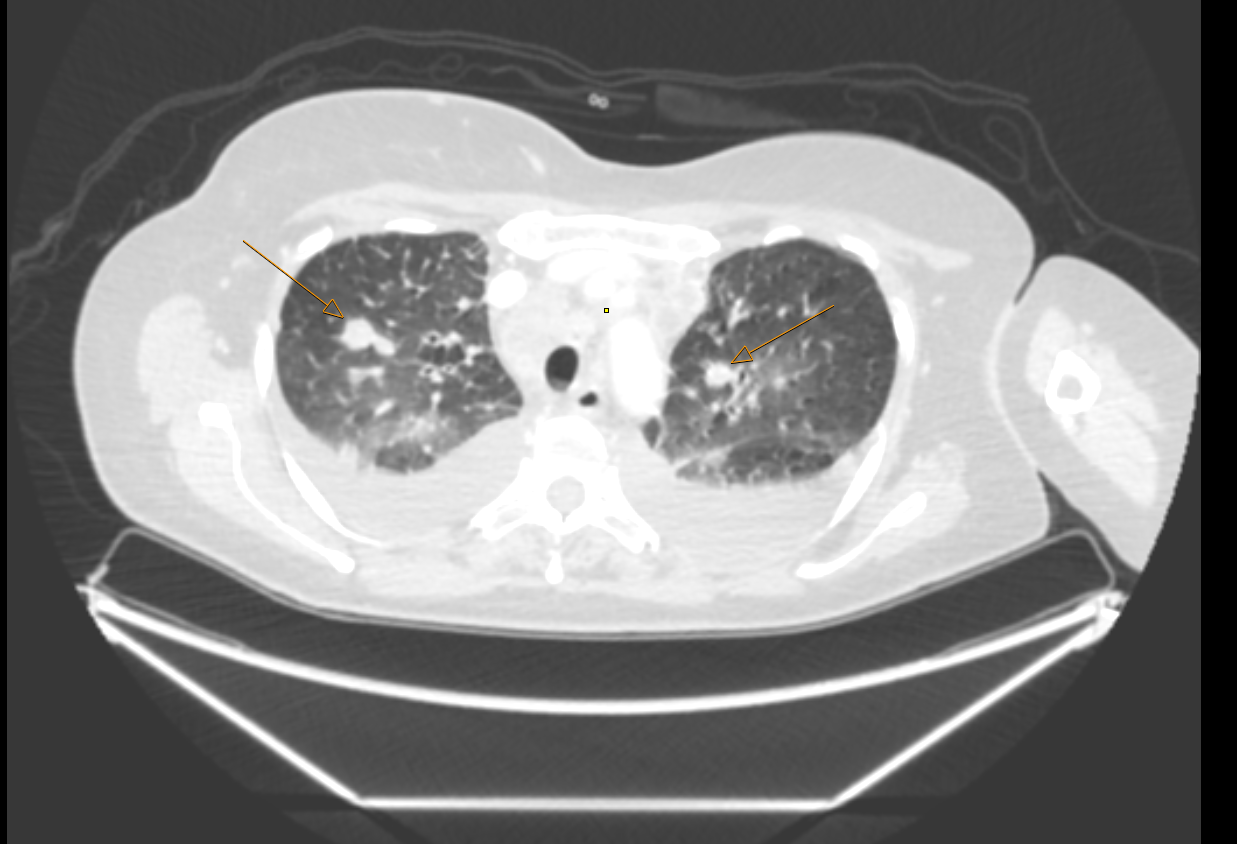  Axial contrast enhanced CT showing multiple nodules (orange arrows) in the upper lobes of both lungs suspect of metastases.  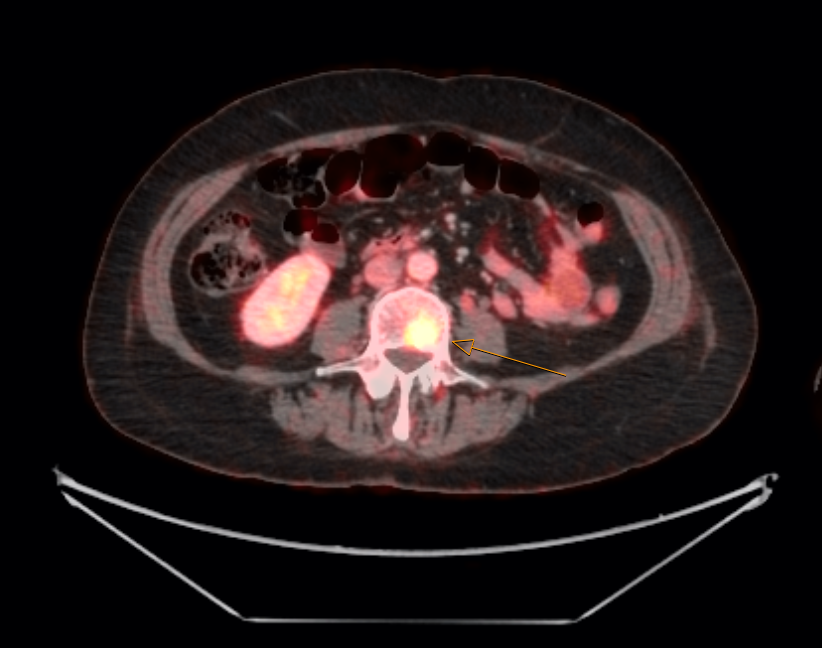  Axial F-18-FDG PET fused to contrast enhanced CT showing a rounded, focally increased FDG-uptake in the vertebral body of L4 (orange arrow) suspect of metastasis.  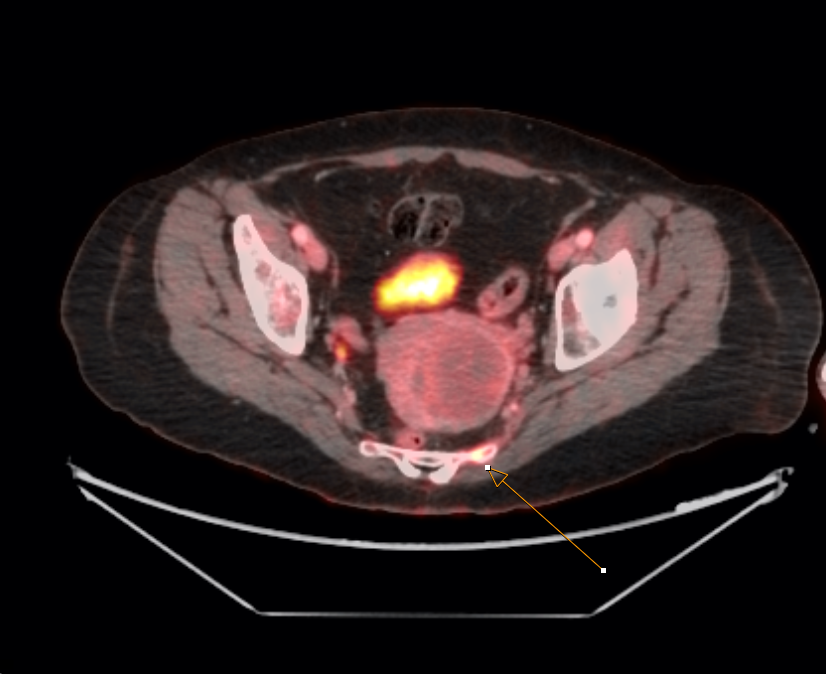  Axial F-18-FDG PET fused to contrast enhanced CT showing focally increased FDG-uptake in the left side of the sacral bone (orange arrow) suspect of metastasis.  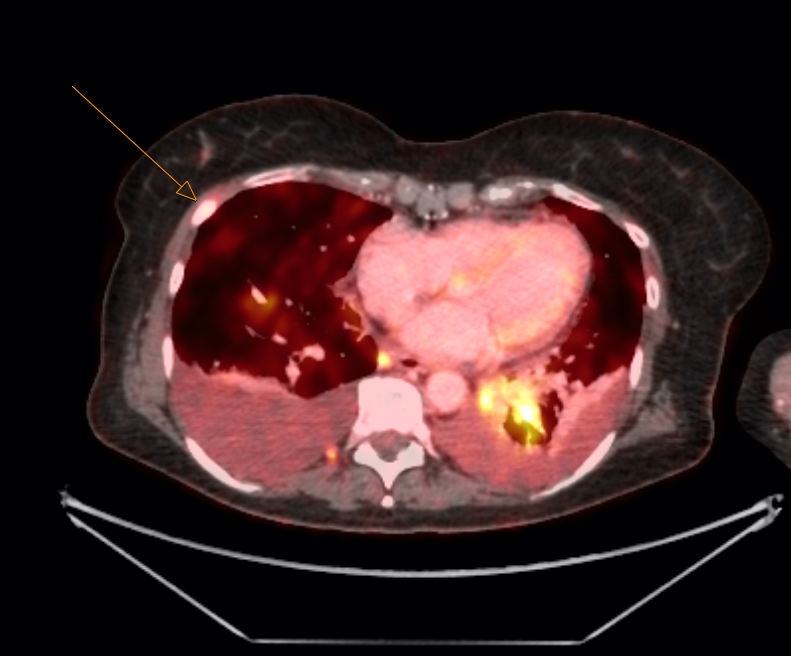  Axial F-18-FDG PET fused to contrast enhanced CT showing focally increased FDG-uptake in the anterior part of the right 6th rib (orange arrow) suspect of metastasis. |

**Supplementary Table 2.** Quality control results for targeted DNA sequencing data (TSO500)

| **patient_ID** | **sample_ID** | **time-point** | **M Reads Mapped** | **M Reads** | **% on target** | **Insert Size** | **≥ 10X** | **≥ 30X** | **Median** | **Mean Cov.** | **TMB** |
| --- | --- | --- | --- | --- | --- | --- | --- | --- | --- | --- | --- |
| 1 | 01_06 | Primary tumor | 168,4 | 167,5 | 97,6 | 42 bp | 16,8% | 3,0% | 4.0X | 12.0X | 151,03 |
| 1 | 01_07 | Lymph node metastasis | 167,1 | 159,5 | 99 | 52 bp | 99,5% | 97,4% | 158.0X | 235.9X | 401,55 |
| 3 | 01_21 | Brain recurrence | 159,7 | 140,6 | 98,5 | 71 bp | 99,7% | 98,9% | 253.0X | 326.7X | 62,37 |
| 3 | 01_20 | Lymph node metastasis | 130,6 | 128,1 | 95,4 | 87 bp | 99,9% | 99,6% | 410.0X | 413.1X | 65,46 |
| 3 | 01_19 | Brain recurrence | 165,4 | 153,1 | 95,8 | 55 bp | 99,5% | 97,7% | 195.0X | 252.5X | 61,86 |
| 6 | 01_15 | Primary tumor | 175,2 | 171,7 | 97,1 | 40 bp | 99,1% | 95,9% | 208.0X | 242.7X | 188,14 |
| 6 | 01_17 | Bone metastasis | 165,6 | 166,5 | 91,2 | 35 bp | 88,2% | 62,9% | 42.0X | 61.2X | 163,92 |
| 6 | 01_16 | Brain recurrence | 138,7 | 139,3 | 88 | 37 bp | 59,2% | 25,5% | 13.0X | 24.4X | 126,8 |
| 7 | 01_04 | Primary tumor | 167,1 | 144,3 | 98,9 | 66 bp | 99,7% | 99,3% | 485.0X | 621.2X | 64,43 |
| 7 | 01_05 | Lymph node metastasis | 175,1 | 164,1 | 99,2 | 61 bp | 99,7% | 98,4% | 169.0X | 205.1X | 63,92 |
| 8 | 01_10 | Primary tumor | 148,2 | 144,2 | 96,7 | 75 bp | 99,6% | 99,2% | 244.0X | 268.3X | 93,81 |
| 8 | 01_11 | Lymph node metastasis | 129,9 | 126,5 | 98,4 | 91 bp | 99,6% | 99,3% | 484.0X | 484.7X | 93,81 |
| 9 | 01_08 | Primary tumor | 148,4 | 146,2 | 99,2 | 88 bp | 99,9% | 99,5% | 371.0X | 375.0X | 66,49 |
| 9 | 01_09 | Liver metastasis | 182,1 | 170,2 | 97,5 | 66 bp | 99,8% | 99,2% | 278.0X | 315.2X | 65,98 |
| 10 | 02_01 | Primary tumor | 135,4 | 135 | 98 | 109 bp | 99,5% | 98,9% | 334.0X | 334.2X | 76,8 |
| 11 | 02_03 | Primary tumor | 165,9 | 154,5 | 99,1 | 70 bp | 99,5% | 98,3% | 220.0X | 258.0X | 500,52 |
| 12 | 02_05 | Primary tumor | 141,2 | 139,8 | 98,4 | 104 bp | 99,8% | 99,2% | 326.0X | 334.4X | 103,61 |
| 13 | 02_15 | Brain recurrence | 171,4 | 164,8 | 99,5 | 79 bp | 98,7% | 92,8% | 104.0X | 135.1X | 665,98 |
| 15 | 01_12 | Primary tumor | 129,4 | 128,5 | 99,1 | 104 bp | 99,9% | 99,7% | 552.0X | 533.9X | 28,87 |
| 15 | 01_14 | Bone metastasis | 123,2 | 121,2 | 97,4 | 93 bp | 99,8% | 99,4% | 473.0X | 493.8X | 28,87 |
| 15 | 01_13 | Brain recurrence | 124,4 | 122,9 | 98,9 | 99 bp | 99,8% | 99,2% | 291.0X | 293.2X | 28,87 |
| 16 | 02_07 | Primary tumor | 137,8 | 131,8 | 95,3 | 74 bp | 99,3% | 95,7% | 108.0X | 129.9X | 764,95 |
| 17 | 02_09 | Primary tumor | 155,8 | 146 | 98,2 | 67 bp | 99,7% | 98,5% | 189.0X | 218.4X | 396,39 |
| 18 | 02_11 | Primary tumor | 152,7 | 143,9 | 97,8 | 77 bp | 99,2% | 97,2% | 190.0X | 213.9X | 453,61 |
| 19 | 02_13 | Primary tumor | 139,7 | 135 | 98,9 | 84 bp | 99,5% | 98,9% | 282.0X | 297.1X | 354,64 |
| 20 | 01_01 | Primary tumor | 144,4 | 142,8 | 96,6 | 106 bp | 99,6% | 99,3% | 566.0X | 785.3X | 21,13 |
| 20 | 01_03 | Lymph node metastasis | 129,7 | 129,7 | 96,8 | 171 bp | 99,6% | 99,4% | 1467.0X | 1903.3X | 21,65 |
| 20 | 01_02 | Brain recurrence | 156,6 | 155,9 | 96,7 | 106 bp | 99,6% | 99,3% | 423.0X | 415.1X | 21,65 |

**Supplementary Table 3.** Overview of clinically relevant mutations employed in the reconstruction of clonal evolution trees

| Patient | Sample | Timepoint | Gene | Chromosome | Position | Pileup | Status | Purity |
| --- | --- | --- | --- | --- | --- | --- | --- | --- |
| pt03 | 01_21 | brain_recurrent | IDH1 | chr2 | 208248388 | 148 | present | 0,86 |
| pt03 | 01_19 | brain_recurrent | IDH1 | chr2 | 208248388 | 87 | present | 0,84 |
| pt03 | 01_20 | lymph_met | IDH1 | chr2 | 208248388 | 225 | present | 0,8 |
| pt03 | 01_21 | brain_recurrent | PIK3CA | chr3 | 179218307 | 230 | present | 0,86 |
| pt03 | 01_19 | brain_recurrent | PIK3CA | chr3 | 179218307 | 161 | present | 0,84 |
| pt03 | 01_20 | lymph_met | PIK3CA | chr3 | 179218307 | 439 | present | 0,8 |
| pt03 | 01_21 | brain_recurrent | BRAF | chr7 | 140776911 | 84 | not_present | 0,86 |
| pt03 | 01_19 | brain_recurrent | BRAF | chr7 | 140776911 | 66 | not_present | 0,84 |
| pt03 | 01_20 | lymph_met | BRAF | chr7 | 140776911 | 255 | present | 0,8 |
| pt03 | 01_21 | brain_recurrent | NOTCH1 | chr9 | 136518292 | 61 | present | 0,86 |
| pt03 | 01_19 | brain_recurrent | NOTCH1 | chr9 | 136518292 | 89 | present | 0,84 |
| pt03 | 01_20 | lymph_met | NOTCH1 | chr9 | 136518292 | 194 | present | 0,8 |
| pt03 | 01_21 | brain_recurrent | TERT | chr5 | 1295135 | 10 | unknown | 0,86 |
| pt03 | 01_19 | brain_recurrent | TERT | chr5 | 1295135 | 9 | not_present | 0,84 |
| pt03 | 01_20 | lymph_met | TERT | chr5 | 1295135 | 24 | present | 0,8 |
| pt07 | 01_04 | primary_tumor | NF1 | chr17 | 31169986 | 241 | not_present | 0,42 |
| pt07 | 01_05 | lymph_met | NF1 | chr17 | 31169986 | 102 | present | 0,15 |
| pt07 | 01_04 | primary_tumor | SMARCA4 | chr19 | 11059852 | 1743 | present | 0,42 |
| pt07 | 01_05 | lymph_met | SMARCA4 | chr19 | 11059852 | 298 | not_present | 0,15 |
| pt07 | 01_04 | primary_tumor | CUX1 | chr7 | 102196714 | 57 | present | 0,42 |
| pt07 | 01_05 | lymph_met | CUX1 | chr7 | 102196714 | 20 | present | 0,15 |
| pt07 | 01_04 | primary_tumor | BRAF | chr7 | 140753336 | 334 | present | 0,42 |
| pt07 | 01_05 | lymph_met | BRAF | chr7 | 140753336 | 100 | present | 0,15 |
| pt07 | 01_04 | primary_tumor | FGFR1 | chr8 | 38418315 | 251 | present | 0,42 |
| pt07 | 01_05 | lymph_met | FGFR1 | chr8 | 38418315 | 140 | not_present | 0,15 |
| pt08 | 01_10 | primary_tumor | RB1 | chr13 | 48381245 | 143 | present | 0,87 |
| pt08 | 01_11 | lymph_met | RB1 | chr13 | 48381245 | 243 | present | 0,36 |
| pt08 | 01_10 | primary_tumor | SMARCA4 | chr19 | 11018959 | 140 | not_present | 0,87 |
| pt08 | 01_11 | lymph_met | SMARCA4 | chr19 | 11018959 | 261 | present | 0,36 |
| pt08 | 01_10 | primary_tumor | CIC | chr19 | 42293758 | 100 | present | 0,87 |
| pt08 | 01_11 | lymph_met | CIC | chr19 | 42293758 | 193 | not_present | 0,36 |
| pt08 | 01_10 | primary_tumor | MAP3K1 | chr5 | 56881760 | 176 | not_present | 0,87 |
| pt08 | 01_11 | lymph_met | MAP3K1 | chr5 | 56881760 | 314 | present | 0,36 |
| pt09 | 01_08 | primary_tumor | DDR2 | chr1 | 162766054 | 216 | not_present | 0,84 |
| pt09 | 01_09 | liver_met | DDR2 | chr1 | 162766054 | 389 | present | 0,73 |
| pt09 | 01_08 | primary_tumor | EGFR | chr7 | 55165319 | 235 | present | 0,84 |
| pt09 | 01_09 | liver_met | EGFR | chr7 | 55165319 | 234 | not_present | 0,73 |
| pt09 | 01_08 | primary_tumor | TERT | chr5 | 1295113 | 16 | present | 0,84 |
| pt09 | 01_09 | liver_met | TERT | chr5 | 1295113 | 9 | not_present | 0,73 |
| pt15 | 01_12 | primary_tumor | TP53 | chr17 | 7673776 | 363 | not_present | 0,32 |
| pt15 | 01_13 | brain_recurrent | TP53 | chr17 | 7673776 | 44 | not_present | 0,79 |
| pt15 | 01_14 | bone_met | TP53 | chr17 | 7673776 | 239 | present | 0,92 |
| pt15 | 01_12 | primary_tumor | PIK3CA | chr3 | 179218303 | 453 | present | 0,32 |
| pt15 | 01_13 | brain_recurrent | PIK3CA | chr3 | 179218303 | 282 | present | 0,79 |
| pt15 | 01_14 | bone_met | PIK3CA | chr3 | 179218303 | 470 | present | 0,92 |
| pt15 | 01_12 | primary_tumor | TERT | chr5 | 1295113 | 38 | not_present | 0,32 |
| pt15 | 01_13 | brain_recurrent | TERT | chr5 | 1295113 | 17 | unknown | 0,79 |
| pt15 | 01_14 | bone_met | TERT | chr5 | 1295113 | 24 | present | 0,92 |
| pt20 | 01_01 | primary_tumor | PTEN | chr10 | 87933061 | 284 | present | 0,88 |
| pt20 | 01_02 | brain_recurrent | PTEN | chr10 | 87933061 | 333 | not_present | 0,56 |
| pt20 | 01_03 | lymph_met | PTEN | chr10 | 87933061 | 819 | present | 0,58 |
| pt20 | 01_01 | primary_tumor | SMARCA4 | chr19 | 11003392 | 374 | not_present | 0,88 |
| pt20 | 01_02 | brain_recurrent | SMARCA4 | chr19 | 11003392 | 232 | not_present | 0,56 |
| pt20 | 01_03 | lymph_met | SMARCA4 | chr19 | 11003392 | 1096 | present | 0,58 |
| pt20 | 01_01 | primary_tumor | TERT | chr5 | 1295113 | 29 | present | 0,88 |
| pt20 | 01_02 | brain_recurrent | TERT | chr5 | 1295113 | 33 | not_present | 0,56 |
| pt20 | 01_03 | lymph_met | TERT | chr5 | 1295113 | 55 | present | 0,58 |

| **Sample** | **Total Reads** | **Mapped Passed** | **Error Rate** | **Non Primary Alignments** | **Reads Mapped** | **Reads Mapped Percent** | **Reads Properly Paired Percent** | | **Reads MQ0 percent** |
| --- | --- | --- | --- | --- | --- | --- | --- | --- | --- |
| GBM_12_Rec | 56195469 | 56195469 | 0,006550366 | 10218204 | 45977265 | 100 | 99,92632228 | 2,164010843 | |
| GBM_12_Met | 54836480 | 54836480 | 0,006750427 | 9027853 | 45808627 | 100 | 99,91149484 | 2,010516491 | |
| GBM_17_Pri | 91121989 | 91121989 | 0,008635587 | 15074391 | 76047598 | 100 | 99,95758446 | 1,921783249 | |
| GBM_9_Pri | 91698203 | 91698203 | 0,008174317 | 14214608 | 77483595 | 100 | 99,95440196 | 1,641187402 | |
| GBM_9_Met | 9010138 | 9010138 | 0,01370446 | 7184380 | 1825758 | 100 | 97,33151929 | 48,51552068 | |
| GBM_17_Rec | 13954770 | 13954770 | 0,01416138 | 9656868 | 4297902 | 100 | 98,47669863 | 28,84835438 | |
| GBM_17_Met | 83314381 | 83314381 | 0,007138253 | 10208775 | 73105606 | 100 | 99,9774901 | 1,20427153 | |
| GBM_19_Pri | 94477606 | 94477606 | 0,008151393 | 12337256 | 82140350 | 100 | 99,97825186 | 1,316030428 | |
| GBM_19_Rec | 37067597 | 37067597 | 0,00882527 | 9995933 | 27071664 | 100 | 99,88363479 | 4,012867476 | |
| GBM_19_Met | 97929117 | 97929117 | 0,008078691 | 13629926 | 84299191 | 100 | 99,96126297 | 1,424746769 | |
| GBM_13_Rec1 | 81918259 | 81918259 | 0,008042855 | 9021750 | 72896509 | 100 | 99,97874658 | 1,035574968 | |
| GBM_13_Rec2 | 47037544 | 47037544 | 0,009008912 | 13813184 | 33224360 | 100 | 99,6612004 | 4,612281471 | |
| GBM_13_Met | 29724330 | 29724330 | 0,00948777 | 10561223 | 19163107 | 100 | 99,70362322 | 6,236415629 | |
| GBM_8_Pri | 132147095 | 132147095 | 0,007710242 | 19066381 | 113080714 | 100 | 99,91738998 | 1,604748445 | |
| GBM_8_Met | 44550698 | 44550698 | 0,006512043 | 5612245 | 38938453 | 100 | 99,97139845 | 1,416702405 | |
| GBM_12_Pri | 43639266 | 43639266 | 0,006832622 | 9693984 | 33945282 | 100 | 99,83192952 | 3,033702887 | |

**Supplementary Table 4.** Quality control results for RNA sequencing data

**Supplementary Table 5.** Overview of antibodies used in the study

| **Antibody** | **Clone/Vendor** | **Concentration** | **Incubation time and temp.** | **Species/type** | **Detection** | **Instrument** |
| --- | --- | --- | --- | --- | --- | --- |
| CD8 | C8 /144B  Dako | 1:50 | 32 min  37 °C | Mouse  Monoclonal antibody | DISCOVERY ChromoMap DAB Kit | BenchMark Discovery ULTRA |
| FOXP3 | 236A/E7  Thermo Fisher Scientific | 1:50 | 16 min  37 °C  +amp. | Mouse Monoclonal antibody | DISCOVERY ChromoMap DAB Kit | BenchMark Discovery ULTRA |
| CD68 | KP-1  Ventana Medical Systems, Inc | Ready-to-use | 32 min  Room temp. | Mouse  Monoclonal antibody | DISCOVERY ChromoMap DAB Kit | BenchMark Discovery ULTRA |
| OLIG2 | EP112  Ventana Medical Systems, Inc | Ready-to-use | 32 min  Room temp. | Rabbit  Monoclonal antibody | DISCOVERY ChromoMap DAB Kit | BenchMark Discovery ULTRA |
| SOX2 | No clone nr.  Novus Biologicals | 1:100 | 32 min  37 °C | Rabbit  Polyclonal  antibody | DISCOVERY ChromoMap DAB Kit | BenchMark Discovery ULTRA |
| MAP2 | HM-2  Sigma Aldrich | 1:12000 | 20 min.  Room temp. | Mouse  Monoclonal  antibody | EnVision FLEX, High pH | Dako Omnis |
| *Abbreviations: CD8* Clusters of differentiation 8; *FOXP3* Forkhead box P3; *CD68* Cluster of differentiation 68; *OLIG2* Oligodendrocyte transcription factor 2; *SOX2* SRY-box transcription factor 2; *MAP2* microtubule-associated protein 2; *DAB* 3,3’-diaminobenzidin; *IHC* Immunohistochemistry. | | | | | | |

| Supplementary Table 6. Classification of tumors according to the 2021 WHO Classification of Tumours of the Central Nervous System^2^ and the DKFZ brain tumor classifier version 12.5^3^ | | | | | | | |
| --- | --- | --- | --- | --- | --- | --- | --- |
| Patient | Tumor incidence | 2021 WHO classification | Family | Family calibrated score | Class | Class calibrated score | Estimated tumor purity  (*InfiniumPurify*) |
| Patients developing true metastases | | | | | | | |
| 1 | Patient excluded during quality control | | | | | | |
| 3 | First recurrence* | Oligodendroglioma, IDH-mutant & 1p/19q codeleted | Diffuse glioma, IDH mutant | 0.99968 | Diffuse glioma, IDH-mutant and 1p19q co-deleted [oligodendroglial type] | 0.99679 | 0.85737 |
| 3 | Second recurrence | Oligodendroglioma, IDH-mutant & 1p/19q codeleted | Diffuse glioma, IDH mutant | 0.90624 | Diffuse glioma, IDH-mutant and 1p19q retained [astroglial type], high grade | 0.81772 | 0.83782 |
| 3 | Metastasis | Oligodendroglioma, IDH-mutant & 1p/19q codeleted | Diffuse glioma, IDH mutant | 0.80889 | Diffuse glioma, IDH-mutant and 1p19q retained [astroglial type], high grade | 0.62301 | 0.79791 |
| 6 | Patient excluded during quality control | | | | | | |
| 7 | Primary tumor | GBM, IDH-WT | Glioblastoma, IDH-wildtype | 0.79388 | Glioblastoma, IDH-wildtype, mesenchymal type | 0.79195 | 0.42220 |
| 7 | Metastasis | GBM, IDH-WT | Unclassified | - | Unclassified | - | 0.15127 |
| 8 | Primary tumor | GBM, IDH-WT | Glioblastoma, IDH-wildtype | 0.97512 | Glioblastoma, IDH-wildtype, RTK2 type | 0.96086 | 0.86916 |
| 8 | Metastasis | GBM, IDH-WT | Glioblastoma, IDH-wildtype | 0.99657 | Glioblastoma, IDH-wildtype, mesenchymal type | 0.99651 | 0.35688 |
| 9 | Primary tumor | GBM, IDH-WT | Glioblastoma, IDH-wildtype | 0.99306 | Glioblastoma, IDH-wildtype, RTK2 type | 0.98291 | 0.83894 |
| 9 | Metastasis | GBM, IDH-WT | Glioblastoma, IDH-wildtype | 0.81409 | Glioblastoma, IDH-wildtype, RTK2 type | 0.57740 | 0.72613 |
| 12 | Primary tumor | GBM, IDH-WT | Glioblastoma, IDH-wildtype | 0.99956 | Glioblastoma, IDH-wildtype, mesenchymal type | 0.99954 | 0.47411 |
| 12 | Metastasis | GBM, IDH-WT | Glioblastoma, IDH-wildtype | 0.94818 | Glioblastoma, IDH-wildtype, mesenchymal type | 0.92883 | 0.61920 |
| 15 | Primary tumor | GBM, IDH-WT | Glioblastoma, IDH-wildtype | 0.62451 | Glioblastoma, IDH-wildtype, mesenchymal type | 0.62440 | 0.32500 |
| 15 | First recurrence | GBM, IDH-WT | Glioblastoma, IDH-wildtype | 0.99809 | Glioblastoma, IDH-wildtype, mesenchymal type | 0.99798 | 0.78610 |
| 15 | Metastasis | GBM, IDH-WT | Glioblastoma, IDH-wildtype | 0.99372 | Glioblastoma, IDH-wildtype, RTK2 type | 0.88625 | 0.92156 |
| 16 | Primary tumor | GBM, IDH-WT | Glioblastoma, IDH-wildtype | 0.85415 | Glioblastoma, IDH-wildtype, RTK2 type | 0.69295 | 0.72542 |
| 16 | Metastasis | GBM, IDH-WT | Glioblastoma, IDH-wildtype | 0.89758 | Glioblastoma, IDH-wildtype, mesenchymal type | 0.88810 | 0.82223 |
| 20 | Primary tumor | GBM, IDH-WT | Glioblastoma, IDH-wildtype | 0.99998 | Glioblastoma, IDH-wildtype, RTK2 type | 0.99998 | 0.88142 |
| 20 | First recurrence | GBM, IDH-WT | Control brain tissues | 0.98664 | Control tissue, hemispheric cortex | 0.98526 | 0.55509 |
| 20 | Metastasis | GBM, IDH-WT | Glioblastoma, IDH-wildtype | 0.95394 | Glioblastoma, IDH-wildtype, mesenchymal type | 0.90587 | 0.61726 |
| Patients developing extracranial extensions | | | | | | | |
| 10 | Primary tumor | GBM, IDH-WT | Glioblastoma, IDH-wildtype | 0.99197 | Glioblastoma, IDH-wildtype, mesenchymal type | 0.99186 | 0.44437 |
| 10 | Scalp lesion | GBM, IDH-WT | Glioblastoma, IDH-wildtype | 0.98611 | Glioblastoma, IDH-wildtype, mesenchymal type | 0.98592 | 0.54506 |
| 11 | Primary tumor | GBM, IDH-WT | Glioblastoma, IDH-wildtype | 0.98722 | Glioblastoma, IDH-wildtype, mesenchymal type | 0.98669 | 0.42096 |
| 11 | Scalp lesion | GBM, IDH-WT | Glioblastoma, IDH-wildtype | 0.46620 | Glioblastoma, IDH-wildtype, mesenchymal type | 0.46119 | 0.16041 |
| 13 | First recurrence* | GBM, IDH-WT | Low-grade ganglioglial/neuroepithelial tumour | 0.37993 | Ganglioglioma | 0.24563 | 0.61718 |
| 13 | Scalp lesion | GBM, IDH-WT | Glioblastoma, IDH-wildtype | 0.98697 | Glioblastoma, IDH-wildtype, RTK2 type | 0.97109 | 0.86216 |
| 17 | Primary tumor | GBM, IDH-WT | Glioblastoma, IDH-wildtype | 0.84426 | Glioblastoma, IDH-wildtype, mesenchymal type | 0.84318 | 0.33696 |
| 17 | Scalp lesion | GBM, IDH-WT | Glioblastoma, IDH-wildtype | 0.99970 | Glioblastoma, IDH-wildtype, mesenchymal type | 0.99969 | 0.40980 |
| 18 | Primary tumor | GBM, IDH-WT | Glioblastoma, IDH-wildtype | 0.86391 | Glioblastoma, IDH-wildtype, mesenchymal type | 0.85025 | 0.62501 |
| 18 | Scalp lesion | GBM, IDH-WT | Glioblastoma, IDH-wildtype | 0.83190 | Glioblastoma, IDH-wildtype, mesenchymal type | 0.81531 | 0.67012 |
| 19 | Primary tumor | GBM, IDH-WT | Glioblastoma, IDH-wildtype | 0.99082 | Glioblastoma, IDH-wildtype, mesenchymal type | 0.99008 | 0.58512 |
| 19 | Scalp lesion | GBM, IDH-WT | Malignant peripheral nerve-sheath tumour | 0.99944 | Malignant peripheral nerve sheath tumour [typical type] | 0.99860 | 0.43982 |
| *Abbreviations: WHO* World Health Organization; *DKFZ* Deutsches Krebsforschungszentrum (German Cancer Research Center); *IDH* Isocitrate dehydrogenase; *GBM* Glioblastoma; *WT* Wildtype; *RTK2* Receptor tyrosine kinase 2. *Tumor material from the first intracranial recurrence was used as there was not enough tissue for DNA extraction or tissue from the primary tumor was unavailable. | | | | | | | |

| **Supplementary Table 7.** Statistics on selected patient characteristics | | | | |
| --- | --- | --- | --- | --- |
| **Variable** | **All patients**  **(*n* = 16)** | **True metastasis**  **(*n* = 10)** | **Extracranial extension**  **(*n* = 6)** | ***P*-value true metastasis vs. extracranial extension** |
| **Gender, *n* (%)** |  |  |  |  |
| Male | 12 (75%) | 8 (80%) | 4 (67%) | 0.6044 |
| Female | 4 (25%) | 2 (20%) | 2 (33%) |  |
| **Age at debut (years), median (range)** | 56.5 (27-65) | 54.5 (27-62) | 59 (40-65) | 0.4375 |
| **IDH1 status*, n (%)*** |  |  |  |  |
| Wildtype | 14 (88%) | 8 (80%) | 6 (100%) | 0.5000 |
| Mutated | 2 (13%) | 2 (20%) | 0 (0%) |  |
| **MGMT status, *n* (%)** |  |  |  |  |
| Methylated | 4 (25%) | 4 (40%) | 0 (0%) | 0.2335 |
| Unmethylated | 12 (75%) | 6 (60%) | 6 (100%) |  |
| **PG or SG, *n* (%)** |  |  |  |  |
| Yes | 6 (43%) | 2 (25%) | 4 (67%) | 0.2774 |
| No | 8 (57%) | 6 (75%) | 2 (33%) |  |
| Not relevant | 2 |  |  |  |
| **Number of craniotomies excl. autopsies, median (range)** | 2 (1-5) | 2 (1-3) | 3 (1-5) | **0.0450** |
| **TMZ plus RT, *n* (%)** |  |  |  |  |
| Yes | 12 (86%) | 6 (75%) | 6 (100%) | 0.4725 |
| No | 2 (14%) | 2 (25%) | 0 (0%) |  |
| Missing | 2 |  |  |  |
| **Bevacizumab plus chemotherapy^*^, *n* (%)** |  |  |  |  |
| Yes | 7 (44%) | 3 (30%) | 4 (67%) | 0.3024 |
| No | 9 (56%) | 7 (70%) | 2 (33%) |  |
| Missing | 2 |  |  |  |
| **Time from diagnosis to ECM (months), median (range)** | 11 (5-140) | 7 (5-140) | 14 (5-39) | 0.6712 |
| *P-*values in bold are significant (Level of significance: P < 0.05). The sum of percentages may deviate from 100 due to rounding. *Abbreviations: IDH1* isocitrate dehydrogenase, *MGMT* O6-methylguanine-DNA-methyltransferase, *PG* primary gliosarcoma, *SG* secondary gliosarcoma, *TMZ* temozolomide, *RT* radiation therapy, *ECM* Extracranial metastasis. ^*^Irinotecan or lomustine. | | | | |

| **Supplementary Table 8.** Survival analyses excluding IDH-mutant patients (n = 2) | | | | |
| --- | --- | --- | --- | --- |
|  | **All patients**  **(*n* = 14)** | **True metastasis**  **(*n* = 8)** | **Extracranial extension**  **(*n* = 6)** | ***P*-value true metastasis vs. extracranial extension** |
| **Overall survival (months), median (range)** | 12.5 (5-44) | 7.5 (5-18) | 17.5 (11-44) | **0.0288** |
| **Survival from metastasis (days), median (range)*** | 86 (15-200) | 31 (15-184) | 137 (54-200) | 0.1068 |
| *P-*values in bold are significant (level of significance: P < 0.05.). *Survival from metastasis in patient 9 was 0 days (detected post-mortem) and this data was not included in this calculation (zero values are by default ignored in the Prism software when performing survival analyses). | | | | |

**Supplementary Table 9.** MRI-based characteristics of the brain tumors at the time of initial diagnosis and time of metastasis

|  | Patient | Primary brain scan  MRI of primary glioma | | | | Secondary brain scan  MRI at the time of metastasis | | | |
| --- | --- | --- | --- | --- | --- | --- | --- | --- | --- |
|  |  | **Location** | **Distance to dura**  Contact/  <2mm/  >2mm | **Distance to large vessels**  Contact, which/  No contact | **Distance to ventricles**  Contact/  <2mm/  >2mm | **Brain tumor visible on MRI**  Yes/No | **Dural metastasis**  Proximal (within craniotomy area)/  Distant /  None | **Distance to large vessels**  Contact, which/  No contact/Not relevant | **Distance to ventricles**  Contact/  <2mm/  >2mm/Not relevant |
| Patients developing true metastases  (No connection to a synchronous brain tumor) | 1 | L temporal | N/A | N/A | N/A | N/A | N/A | N/A | N/A |
|  | 3* | N/A | N/A | N/A | N/A | Yes | None | Contact, sinus sagittalis superior | >2mm |
|  | 6 | L frontal | Contact | Contact, superior sagittal sinus | >2mm | No | Distant | Not relevant | Not relevant |
|  | 7 | N/A | N/A | N/A | N/A | N/A | N/A | N/A | N/A |
|  | 8 | L occipital | N/A | N/A | N/A | No | None | Not relevant | Not relevant |
|  | 9** | R frontal | Contact | No contact | Contact | No | None | Not relevant | Not relevant |
|  | 12 | L parietooccipital | Contact | No contact | >2mm | Yes | None | No contact | Contact |
|  | 15 | R parietooccipital | <2mm | No contact | >2mm | Yes | None | Contact, sinus sagittalis superior | Contact |
|  | 16 | R frontoparietal | <2mm | Contact, a. cerebri media | >2mm | Yes | Distant | No contact | >2mm |
|  | 20 | R frontal | Contact | Contact, superior sagittal sinus | >2mm | Yes | None | No contact | Contact |
| Patients developing extracranial extensions  (Scalp lesion with connection to a synchronous brain tumor) | 10 | R parietooccipital | <2mm | No contact | Contact | Yes | Proximal  Distant | No contact | Contact |
|  | 11 | R frontal | N/A | N/A | N/A | Yes | Proximal | Contact, anterior cerebral artery | Contact |
|  | 13 | L tempoparietal | Contact | No contact | Contact | Yes | None | No contact | Contact |
|  | 17 | R temporal | Contact | No contact | >2mm | Yes | Proximal | No contact | Contact |
|  | 18 | L frontal | >2mm | Contact, middle cerebral artery | >2mm | Yes | Distant | Contact, a. cerebri media | Contact |
|  | 19 | R temporal | Contact | No contact | >2mm | Yes | None | Contact, transverse and sigmoid sinus | Contact |
| *No MRI available from the time of detection of metastasis, instead an MRI of the recurrent tumor obtained five months prior to detection of metastasis was used. ** No MRI available from the time of detection of metastasis, instead an MRI of the recurrent tumor obtained four months prior to detection of metastasis was used. *Abbreviation*s: *MRI* magnetic resonance imaging, *L* left, *R* right, *N/A* Not available. | | | | | | | | | |

**Supplementary Table 10.** Base MRI characteristics of the tumor cohort along with volumetry results

| **Patient** | **Location** | **Contrast-enhancement pattern** | **Central necrosis** | **FLAIR/T2-mismatch** | **Mass effect** | **Total tumor volume, cm^3^** | **Contrast-enhancement volume, cm^3^** | **Necrosis volume, cm^3^** | **FLAIR volume, cm^3^** |
| --- | --- | --- | --- | --- | --- | --- | --- | --- | --- |
| 1 |  | N/A | N/A | N/A | N/A | N/A | N/A | N/A | N/A |
| 3 |  | N/A | N/A | N/A | N/A | N/A | N/A | N/A | N/A |
| 6 | L frontal | None | No | N/A | Yes | N/A | N/A | N/A | N/A |
| 7 |  | N/A | N/A | N/A | N/A | N/A | N/A | N/A | N/A |
| 8 |  | N/A | N/A | N/A | N/A | N/A | N/A | N/A | N/A |
| 9 | R frontal | Solid | Yes | No | Yes | 53.4 | 33.6 | 4.6 | 208.4 |
| 10 | R parietooccipital | Rim | Yes | No | Yes | 69.0 | 25.2 | 45.1 | 102.8 |
| 11 |  | N/A | N/A | N/A | N/A | N/A | N/A | N/A | N/A |
| 12 | L parietooccipital | Rim | Yes | No | Yes | 25.5 | 11.3 | 10.2 | 69.1 |
| 13 | L temporoparietal | Mixed | Yes | No | Yes | 95.2 | 43.2 | 43.2 | 87.1 |
| 15 | R parietooccipital | Mixed | Yes | No | Yes | 56.4 | 26.7 | 13.3 | 157.2 |
| 16 | R frontoparietal | Rim | Yes | No | Yes | 13.5 | 4.5 | 7.1 | 64.9 |
| 17 | R temporal | Mixed | Yes | No | Yes | 104.4 | 37.6 | 56.6 | 91.2 |
| 18 | L frontal | Rim | Yes | No | Yes | 28.3 | 12.1 | 9.9 | 170.9 |
| 19 | R temporal | Rim | Yes | No | Yes | 22.0 | 12.4 | 3.2 | 60.7 |
| 20 | R frontal | Solid | Yes | No | Yes | 42.1 | 30.0 | 6.7 | 96.8 |
| *Abbreviation*s: *MRI* magnetic resonance imaging, *L* left, *R* right, *N/A* Not available. | | | | | | | | | |

| **Supplementary Table 11.** Glioma-specific genetic and prognostic markers in the tumors obtained from visual inspection of copy-number alteration plots derived from the online DKFZ brain tumor classifier version 12.5^3^ | | | | | | |
| --- | --- | --- | --- | --- | --- | --- |
| **Patient** | **Tumor**  **incidence** | ***1p/19q* codeletion** | ***CDKN2A/B* loss (homozygous)** | **+7/-10**  **one or both** | ***EGFR* amplification** | **Comments/other findings** |
| **3** | First recurrence* | Yes | No | Yes | No | **Gain:** 7  **Loss**: 13q |
|  | Recurrent | Yes | Ambiguous | Yes | No | Not clear if homo- or heterozygous loss of CDKN2A/B  **Gain**: 1q, 7, 8, 11, 17  **Loss**: 4 |
|  | Metastasis | Yes | Yes | Yes | No | **Gain**: 1q, focal 3q, 7, 8, focal 9p, 9q, 12q, 17, 19p  **Loss**: 4q, 11q |
| **8** | Primary | No | No | Ambiguous | No | Not clear if loss of 10 |
|  | Metastasis | No | No | Yes | No | **Gain**: 7  **Loss**: 10 |
| **9** | Primary | No | Yes | Yes | No | **Gain**: 7, 9q, focal 19p, focal 19q  **Loss**: Focal 14q, focal 17q, focal 18p, 22q |
|  | Metastasis | No | Yes | Yes | No | **Gain**: 7, 9q, focal 19q  **Loss**: Focal 1p, 14q, 17q, 18p, 19p, 22 |
| **7** | Primary | No | Ambiguous | Yes | No | Possible homozygous CDKNA2A/B deletion  **Gain**: 7, 19, 20  **Loss**: 22q |
|  | Metastasis | No | No | Yes | No | **Gain:** 7 |
| **15** | Primary | No | No | Yes | No | Not clear if gain of 7  **Loss**: 6, 10, 13q, 14q, 22q (?) |
|  | Recurrent | No | Yes | Yes | No | **Gain**: Focal 1q, 1p, focal 3q, 5, 6, 7, 9(?), 13q, focal 15q, 17, 20, 21 |
|  | Metastasis | No | Yes | Yes | No | Baseline difficult to establish  **Gain**: 7  **Loss**: 10 |
| **20** | Primary | No | No | Yes | Yes | **Gain**: 7, 12q, 14q, 19, 20  **Loss**: Focal 2q, focal 3q, focal 6q, 10, 11, focal 15q, 16q, focal 22q |
|  | Recurrent | - | - | - | - | Flat profile |
|  | Metastasis | No | No | Yes | Yes | Focal EGFR ampl.  **Gain**: 7, 8, focal 12q (CDK4, MDM2), 14q, 19, 20  **Loss**: focal 2q, focal 3q, focal 6q, 10, 11, 13q, 21q, 22q |
| **10** | Primary | No | No | Yes | No | **Gain**: focal 3q, 7  **Loss**: 10, focal 13q |
|  | Metastasis | No | No | Yes | No | Possibly ampl. of CDKN2A/B  **Gain**: 1p, focal 1q  **Loss**: 3p, 4p, focal 4q, 10, 11, focal 13q, 17, 18q, 19 (?) |
| **11** | Primary | No | Yes | Yes | No | PDGFRA ampl.,  Heterozygous TP53 loss  **Gain**: focal 6p, 7, 14q (?)  **Loss**: 9, 10, 13q, 15q, 18 |
|  | Metastasis | No | No | Ambiguous | No | Difficult to interpret profile  Possibly gain of 7/loss of 10 |
| **12** | Primary | No | No | Yes | No | **Gain:** 7  **Loss**: 4q, focal 5p, focal 9p, 10, 12p, 13q, focal 14q, 16q, 18, focal 19q, 22q |
|  | Metastasis | No | No | Yes | No | **Gain:** 7  **Loss**: focal 4q, focal 9p, 10, 12p, 13q, focal 14q, 16q, 18, focal 19q, 22q |
| **13** | First recurrence* | No | No | Yes | No | **Gain**: 7, 19p, focal 19q, 20  **Loss**: 10, 13q, 17p, 17q, focal 19q, 22q |
|  | Metastasis | No | No | Yes | No | **Gain**: 7  **Loss**: 10 |
| **16** | Primary | No | Yes | Yes | No | **Gain**: 2p, 7, 19  **Loss**: 6, 10, 13q, 14q, focal 17q |
|  | Metastasis | No | Yes | Yes | No | **Loss**: 10 |
| **17** | Primary | No | No | Yes | No | **Gain**: 1, 3, 7  **Loss**: 8, 10, 12, 13q, 14q, 22q |
|  | Metastasis | No | No | Yes | No | **Gain**: 1, 3, 6p, focal 6q, 7, 17, 21q  **Loss**: 2, 4, focal 6q, 8, 10, 11 (?), 22q |
| **18** | Primary | No | Yes | Yes | No | **Gain**: 5, 7, 8  **Loss**: focal 2q, focal 3q, 4, 6, 10, 11, 12p, 13q, 14q, focal 16q, 17, 22q |
|  | Metastasis | No | Yes | Yes | No | **Gain**: 1q, 5p, 7, 8, 9p, 9q, 15q, 16p, 20  **Loss**: focal 2q, focal 3q, 3p, focal 4q, 6, 10, 11, 14q, focal 16q, focal 22q (?) |
| **19** | Primary | No | Yes | Yes | No | **Gain:** 7  **Loss**: focal 6q, focal 9p, 10, 13q, focal 17q, 17p, focal 18q |
|  | Metastasis | No | Yes | Yes | No | **Gain**: 4p, 7, focal 8p, 8q, focal 17q  **Loss**: focal 2q, focal 6q, focal 9q, focal 18q |
| *Abbreviations: CDKN2A/B* cyclin-dependent kinase inhibitor 2A/B; *EGFR* Epidermal growth factor receptor; *Ampl* Amplification; *CDK4* Cyclin-dependent kinase 4; *MDM2* Mouse double minute 2 homolog; *PDGFRA* platelet-derived growth factor receptor A; *TP53* Tumor protein p53. Copy number alterations that were uncertain are indicated by a question mark. *Tumor material from the first intracranial recurrence was used as there was not enough tissue for DNA extraction or tissue from the primary tumor was unavailable. | | | | | | |

**Supplementary Figure 1.** MRI scans with examples of different tumor parameters investigated. (**A**) Partly solid and contrast-enhancing, partly cystic tumor with dural contact (arrow). (**B**) Contrast-enhancing tumor surrounding a branch of the right middle cerebral artery (arrow). (**C**) Contrast-enhancing tumor surrounding (arrow) the right occipital horn. (**D**) Nodular contrast enhancement of the meninges just posterior to the craniotomy burr hole, interpreted as dural metastasis. All scans are T1-weighted post-gadolinium axial images. Created in BioRender. Kristensen, B. (2025) <https://BioRender.com/1dj6smv>.

**Supplementary Figure 2.** Variant allele frequency (VAF) distribution. Density plot of the VAF of single nucleotide variants (SNVs), comparing the primary tumors (x-axis) and the recurrences together with the metastasis (y-axis).

**Supplementary Figure 3.** Phylogenetic trees showing the clonal evolution of six tumors in the cohort with nodes representing clones and branches representing evolution paths. Branches are labeled with potential driver mutations and somatic copy number alterations inferred from the copy number alteration (CNA) plots obtained from the DKFZ brain tumor classifier V.12.5^3^. Colored branches indicate divergent evolution, distinguishing each clone from the ancestral one, and the timeline is proportional to the number of days between surgeries. Inferred clonality is based on the variant allele frequency (VAF) of the mutations, with a lower VAF suggesting a subclonal variant. *Abbreviations:* *IDH* Isocitrate dehydrogenase; *Codel* Co-deletion; *IDH1* Isocitrate dehydrogenase 1; *Mut* mutated; *PIK3CA* Phosphatidylinositol-4,5-bisphosphate 3-kinase catalytic subunit alpha; *NOTCH1* Notch receptor 1; *TERT* Telomerase reverse transcriptase; *CDKN2A/B* Cyclin dependent kinase inhibitor 2A/B; *Homodel* Homozygous deletion; *BRAF* B-Raf proto-oncogene, serine/threonine kinase; *CUX1* Cut like homeobox 1; *FGFR1* Fibroblast growth factor receptor 1; *SMARCA4* SWI/SNF related, matrix associated, actin dependent regulator of chromatin, subfamily a, member 4; *NF1* Neurofibromin 1; *TP53* Tumor protein p53; *RB1* RB transcriptional corepressor 1; *CIC* Capicua transcriptional repressor; *MAP3K1* Mitogen-activated protein kinase kinase kinase 1; *EGFR* Epidermal growth factor receptor; *PTEN* Phosphatase and tensin homolog; *DDR2* Discoidin domain receptor tyrosine kinase 2.

**Supplementary Figure 4.** Tumor class predicted scores from the DKFZ brain tumor classifier version 12.5^3^ and tumor purity based on genome-wide DNA methylation data. **(A)** Distribution of DKFZ brain tumor classification scores for all samples demonstrated in a pie chart. **(B)** Estimated tumor purity in primary tumors, recurrences, and metastases using the R-package InfiniumPurify. Boxes show 25^th^ and 75^th^ percentiles and the line in the middle is plotted as the median. Whiskers represent minimum to maximum values. **(C)** Calibrated methylation class scores (Y-axis) correlated significantly with tumor purity (X-axis) (R = 0.3564, *P* = 0.0.0490). Level of significance: *P* < 0.05.

**Supplementary Figure 5.** Estimates of tumor stemness from genome-wide DNA methylation profiling (EPIC arrays) within and between groups of patients developing true metastases and extracranial extensions. Boxes show 25^th^ and 75^th^ percentiles and the line in the middle is plotted as the median. Whiskers represent minimum to maximum values. Level of significance: *P* < 0.05. *Z-transformed. *Abbreviations: mDNAsi* Methylation-based stemness index.

**Supplementary Figure 6.** Profiling of the tumor microenvironment in paired primary brain tumors, intracranial recurrences, and metastases based on automated quantitation of immune cell markers CD8, FOXP3, and CD68, stemness markers SOX2 and OLIG2 and mature neuron marker MAP2. CD8, FOXP3, OLIG2, and MAP2 have been log-transformed to increase visibility. CD8 and MAP2 quantitations contained a zero value not shown due to limitations to the logarithmic scale. Boxes show 25^th^ and 75^th^ percentiles and the line in the middle is plotted as the median. Whiskers represent minimum to maximum values. A minimum of two pairs were required for the paired t-test. Level of significance: *P* < 0.05. Representative histology images are provided from before and after applying the automated quantitation APPs (classifiers) in the Visiopharm software. Histology images are displayed at 20x magnification. *Abbreviations*: *IHC* immunohistochemistry, *CD8* Cluster of differentiation 8; *FOXP3* Forkhead box P3; *CD68* Cluster of differentiation 68; *SOX2* SRY-box transcription factor 2; *OLIG2* Oligodendrocyte transcription factor 2; *MAP2* microtubule-associated protein 2. Created in BioRender. Kristensen, B. (2025) https://BioRender.com/x01e049.

**Supplementary Figure 7.** Relative fractions of B cells, NK cells, CD4 T cells, CD8 T cells, monocytic derived cells, neutrophils, and eosinophils in primary tumors and metastases from cell-type deconvolution analysis. Boxes show 25^th^ and 75^th^ percentiles and the line in the middle is plotted as the median. Whiskers represent minimum to maximum values. A minimum of two pairs were required for the paired t-test. Level of significance: *P* < 0.05. *Abbreviations*: *NK* Natural killer; *CD4* Cluster of differentiation 4; *CD8* Cluster of differentiation 8. *NA* Each pair have the same difference and paired t-test cannot be calculated OR all values are identical in both groups and unpaired t-test cannot be calculated.

**Supplementary Figure 8.** GFAP expression tended to decrease in the metastases. Representative histology images from selected patients in the cohort. Histology images are displayed at 20x magnification. Scale bar = 100 μm. *Abbreviations: GFAP* Glial fibrillary acidic protein. Created in BioRender. Kristensen, B. (2025) <https://BioRender.com/bt7jehc>.

**REFERENCES**

1. Johansen MD, Rochat P, Law I, Scheie D, Poulsen HS, Muhic A. Presentation of Two Cases with Early Extracranial Metastases from Glioblastoma and Review of the Literature. *Case Rep Oncol Med*. 2016;2016. doi:10.1155/2016/8190950

2. WHO Classification of Tumours Editorial Board. *World Health Organization Classification of Tumours of the Central Nervous System*. 5th ed. International Agency for Research on Cancer; 2021.

3. Capper D, Stichel D, Sahm F, et al. Practical implementation of DNA methylation and copy-number-based CNS tumor diagnostics: the Heidelberg experience. *Acta Neuropathol*. 2018;136(2):181-210. doi:10.1007/s00401-018-1879-y
