# Supplementary figures and images for: Clinical, radiological, and molecular insights into extracranial metastases from adult gliomas
Source: Neuro Oncol. 2025 Aug 16;28(1):99–114. doi: 10.1093/neuonc/noaf178 (PMC12962640; doi:10.1093/neuonc/noaf178)

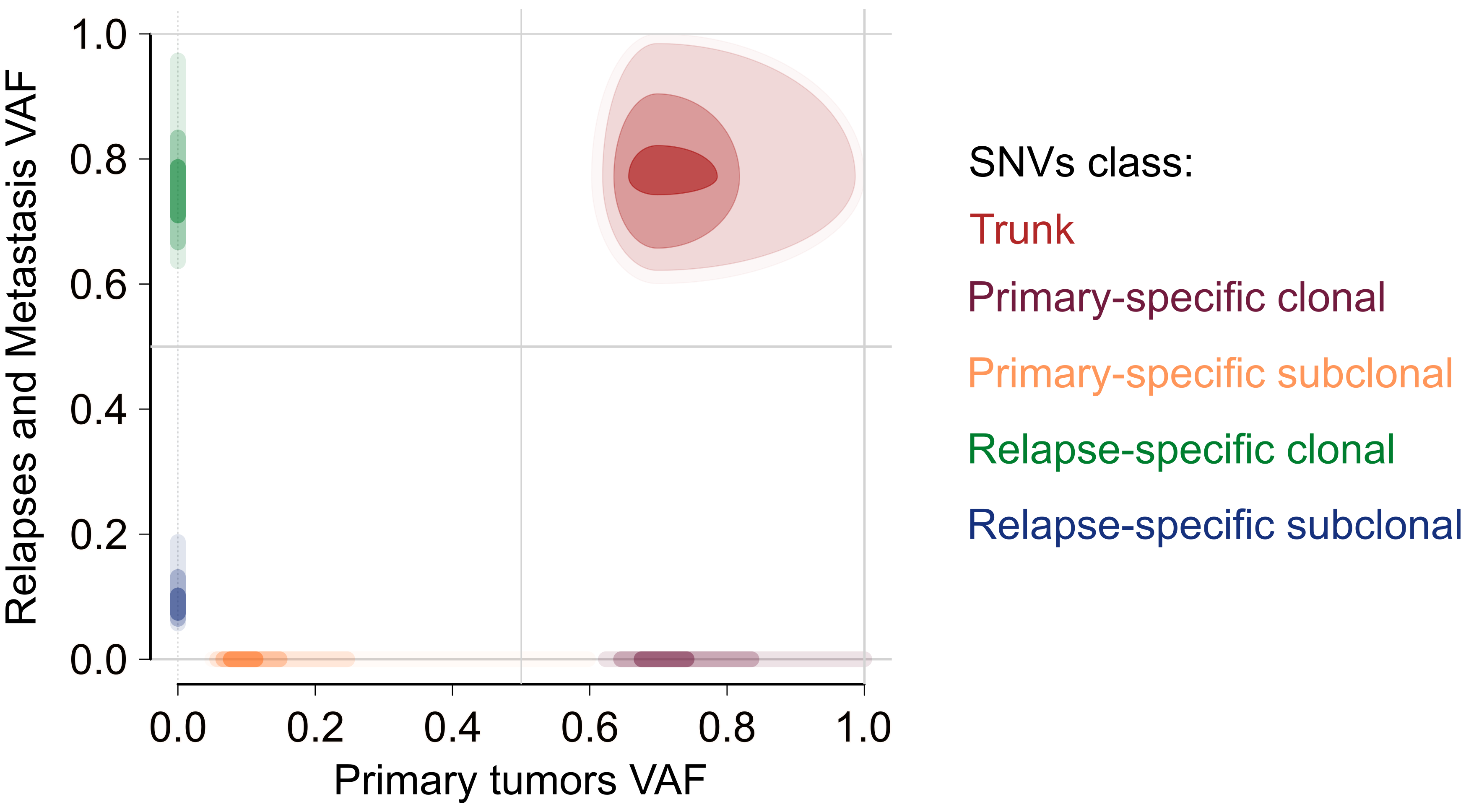

Supplement: noaf178_Supplementary_Data [file noaf178_supplementary_data.zip › Suppl. Fig 2.tiff]

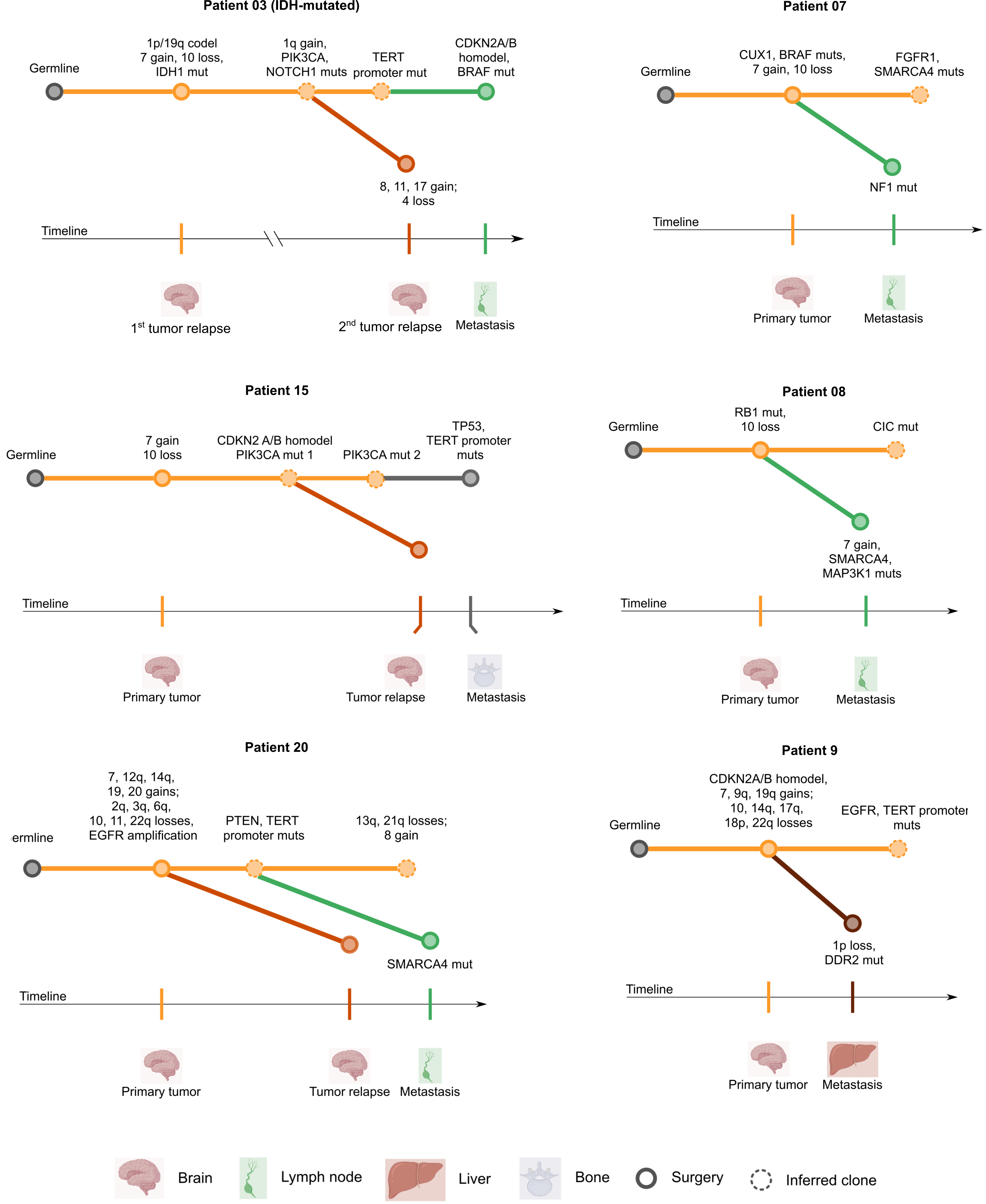

Supplement: noaf178_Supplementary_Data [file noaf178_supplementary_data.zip › Suppl. Fig 3.tiff]

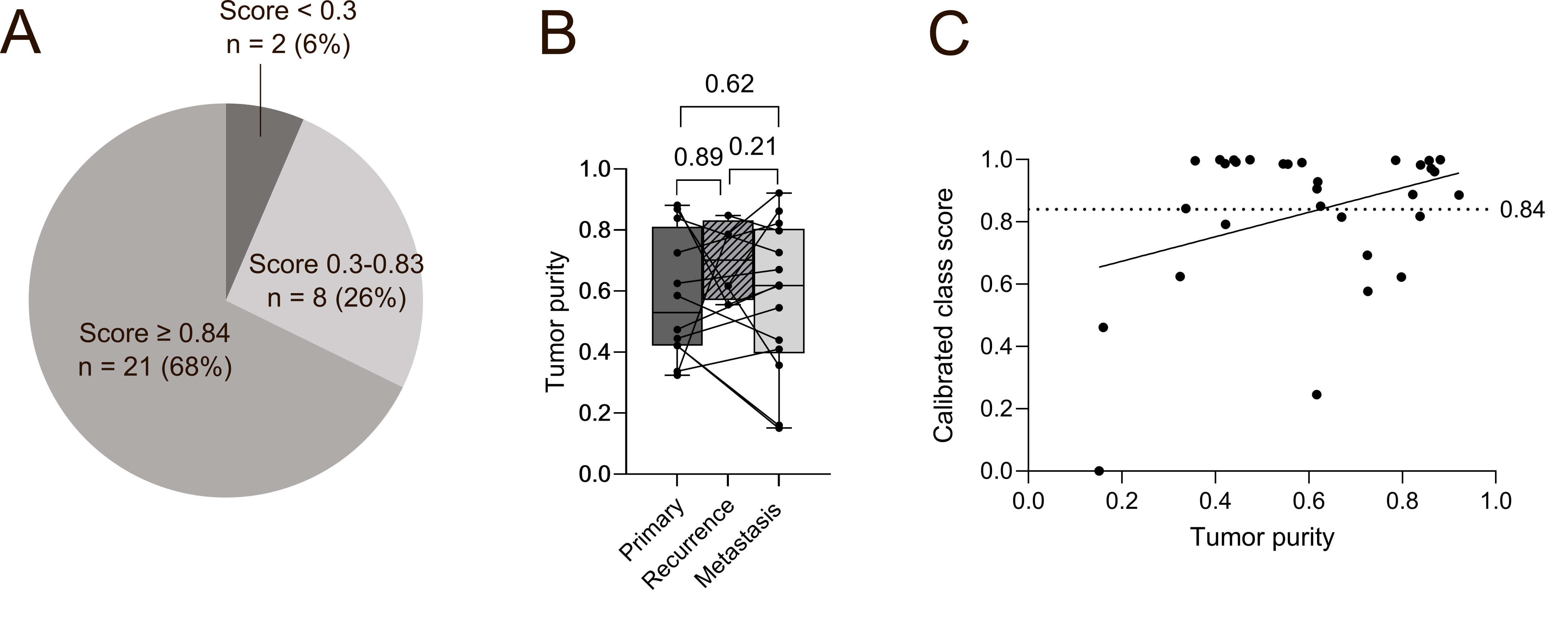

Supplement: noaf178_Supplementary_Data [file noaf178_supplementary_data.zip › Suppl. Fig 4.tiff]
